# Representation and processing of mass and count nouns: a review

**DOI:** 10.3389/fpsyg.2014.00589

**Published:** 2014-06-11

**Authors:** Nora Fieder, Lyndsey Nickels, Britta Biedermann

**Affiliations:** Department of Cognitive Science, ARC Centre of Excellence in Cognition and its Disorders, Macquarie UniversitySydney, NSW, Australia

**Keywords:** lexical-syntax, countability, mass nouns and count nouns, language production, language comprehension

## Abstract

Comprehension and/or production of noun phrases and sentences requires the selection of lexical-syntactic attributes of nouns. These lexical-syntactic attributes include grammatical gender (masculine/feminine/neuter), number (singular/plural) and countability (mass/count). While there has been considerable discussion regarding gender and number, relatively little attention has focused on countability. Therefore, this article reviews empirical evidence for lexical-syntactic specification of nouns for countability. This includes evidence from studies of language production and comprehension with normal speakers and case studies which assess impairments of mass/count nouns in people with acquired brain damage. Current theories of language processing are reviewed and found to be lacking specification regarding countability. Subsequently, the theoretical implications of the empirical studies are discussed in the context of frameworks derived from these accounts of language production (Levelt, 1989; Levelt et al., 1999) and comprehension (Taler and Jarema, 2006). The review concludes that there is empirical support for specification of nouns for countability at a lexical-syntactic level.

“Many of the things you can count don't count. Many of the things you can't count really count.”

(Albert Einstein, 14.03.1879–18.04.1955)

What is the difference between *rice* and *lentils, garlic* and *onions* or *asparagus* and *salsifies*^1^? Rice and lentils are small, similar looking entities which appear in bigger clusters and often are used as a side dish. Garlic and onions belong to the same plant genus *Allium*. Both grow underground and the bulb of the plant is used for cooking due to its spicy flavor. Asparagus and salsifies are also both similar looking vegetables and salsifies are even colloquially referred to as *poor man's asparagus*. All in all, these entities seem to have much in common regarding their origin, appearance, and use. Why then, do we say: “There is an onion.” but “There is some garlic.,” “There are a few lentils left.” but “There is a little rice left.” or “There are too many salsifies.” but “There is too much asparagus.”? Why is it that onion, lentil, and salsifies are count nouns, and garlic, rice and asparagus mass nouns? In other words, why and how do we grammatically distinguish these nouns for countability as English speakers?

Nouns have a variety of lexical-syntactic attributes. These include grammatical gender (e.g., feminine, masculine), number (i.e., singular, plural) and countability (i.e., mass, count). In language production, these attributes determine the form of adjacent constituents in a phrase or a sentence, such as determiners. For example, in German and French, the definite determiner “der_masculine_,” “le_masculine_” (the) is required for singular nouns of masculine gender and “die_feminine_,” “la_feminine_” for singular nouns of feminine gender. For example in the phrases “der_masculine_ Hund_masculine, singular_,” “le_masculine_ chien_masculine, singular_” (the dog) for a singular noun of masculine gender, “die_plural_ Hunde_masculine, plural_,” “les_plural_ chiens_masculine, plural_” (the dogs) for a plural noun of masculine gender; similarly, in English and Dutch while the indefinite (a, een) and definite determiners (the, de/het) are acceptable for count nouns, only the definite determiner can be used for mass nouns (e.g., ^*^a honey, ^*^een honing). Therefore to produce grammatical sentences or to fully understand a sentence, information regarding countability needs to be activated and retrieved from the mental lexicon.

There has been a relatively large amount of attention in the literature on some lexical-syntactic attributes, including number (e.g., Baayen et al., 1996, 1997; Bock et al., 2001, 2004, 2012; Schiller and Caramazza, 2002; Sonnenstuhl and Huth, 2002; Bock and Middleton, 2011) and grammatical gender (Schriefers, 1993; Badecker et al., 1995; Van Berkum, 1997; Vigliocco et al., 1997; La Heij et al., 1998; Jacobsen, 1999; Jescheniak, 1999; Meyer and Bock, 1999; Vigliocco and Franck, 1999, 2001; Vigliocco and Hartsuiker, 2002). However, countability is an equally valid lexical-syntactic attribute which is distinguished in many languages, but yet has received far less attention. We will therefore review the current literature on countability, both experimental and theoretical.

We will first introduce the fundamental semantic and syntactic characteristics of mass and count nouns in order to understand their linguistic differentiation. Subsequently, three theories will be presented which have explicitly discussed the representation of mass and count nouns (Levelt, 1989; Levelt et al., 1999; Barner and Snedeker, 2005, 2006; Taler and Jarema, 2006). We then go on to review experimental studies which have investigated the representation and processing of mass and count nouns. We first focus on investigations of language processing in adults without language impairment, comprising studies on the availability of grammatical information in the Tip-of-the-Tongue state (ToT), semantic categorization, and grammatical judgment. Subsequently, we review case studies which assess impairments of mass nouns and processing of mass and count nouns in people with language impairment as a result of stroke and progressive neurological disorders roke a impairment language impairmentage and cognitive processes? (e.g., Alzheimer's disease, semantic dementia). Finally, we bring the disparate literatures together and draw some conclusions from the research to date.

## Characteristics of count nouns and mass nouns

Many languages differentiate between count nouns (e.g., chair, dog) and mass nouns (e.g., honey, gold). Mass and count nouns have been argued to differ semantically, syntactically and morphologically. However, there is still disagreement regarding whether the mass/count categorization can be attributed to differences in semantics (e.g., Jackendoff, 1991; Armon-Lotem et al., 2004) or whether it reflects a syntactic distinction (e.g., Shapiro et al., 1989; Vigliocco et al., 1999; Garrard et al., 2004) or both (Warrington and Crutch, 2005). A similar debate is ongoing for the acquisition of conceptual-semantic and lexical-syntactic knowledge about mass and count nouns. Quine (1960) proposed that it is mass and count syntax which provides a means by which children can acquire conceptual-semantic knowledge of physical objects, such as individuation and quantification (syntactic bootstrapping). In contrast, MacNamara (1972, 1982) assumed, however, that it is conceptual-semantic knowledge in form of prelinguistically acquired categories such as “object” and “substance” which leads to the acquisition of the syntactic categories mass and count (semantic bootstrapping) (see also Barner and Snedeker, 2005).

Count nouns and mass nouns differ in ways which can inform ideas about their representation at different levels of language processing. On the one hand, the referents of the two noun classes have been suggested to differ in their semantic and/or conceptual characteristics (Wisniewski et al., 2003; Armon-Lotem et al., 2004). A count noun object does not apply to any of its parts (e.g., table applies to a single table but not to its legs). In other words, count noun referents are indivisible or atomic, and therefore can be sorted and counted. In contrast, many mass noun referents apply to their parts (e.g., the term “water” can apply to an obtainable portion of water like a puddle). They are non-atomic and often represent substances (e.g., water, honey) or aggregates^2^ (e.g., rice, confetti) without defined boundaries. Thus, a combination of two samples of a mass noun referent like, for instance, water plus water would result in one larger sample of water. As this makes it impossible to count or sort mass noun referents, they are mostly measured (e.g., one liter of water, two teaspoons of honey). Count noun referents on the other hand have clear and accessible boundaries. The sum of two chairs would not lead to one bigger chair.

However, the distinction between count and mass nouns is not always conceptual-semantically transparent. Some nouns refer to distinct objects (e.g., broccoli, bread) but are still syntactically mass nouns. Similarly, nouns which are syntactically count nouns can also refer to small, homogenous entities and therefore represent aggregates (e.g., lentils, peas, pearls) just like mass noun referents. In some cases, there are even nouns which refer to the same entities but belong to a different noun category (e.g., pebbles vs. gravel, garments vs. clothing). A conceptual-semantic distinction underlying countability becomes even harder to maintain when abstract noun referents are considered (e.g., abstract count nouns: future, dream, idea vs. abstract mass nouns: appetite, irony, evidence) or in reference to superordinate categories (e.g., countable superordinate categories: vegetable, animal vs. non-countable superordinate categories: clothing, furniture) (Middleton et al., 2004).

The lack of conceptual-semantic transparency between mass and count categories is also reflected in cross-linguistic differences regarding categorization. For example, some mass noun referents in English, are labeled as count nouns in other languages. For example, “bread” and “soup” are referred to using count nouns in German (Brot, Suppe), “spinach” and “spaghetti” using count nouns in Italian (spinaci, spaghetti) and “furniture” and “information” using count nouns in French (meuble, information). Hence, the categorization of some noun referents as count or mass is language-specific. Indeed, some languages do not even have this distinction. For instance, in Japanese all nouns are neutral regarding countability (Iwasaki et al., 2010).

The distinction between mass and count nouns can also depend on the context. The same noun referent (e.g., coffee, tea) which is conceptual-semantically classified as a mass noun can often be used in another context as a count noun (e.g., Three coffees, please.) by deleting the unit of measurement (e.g., “cups of”) from the surface structure. Furthermore, the same noun can be used sometimes with either mass or count syntax without any deletion in the surface structure [e.g., I'll go buy a cake (count). vs. I want cake (mass) for dessert.]. Taler and Jarema (2007) referred to this group of nouns as dual nouns.

From this brief overview of the conceptual-semantic differences between mass and count noun referents, it is clear that the distinction between the categories is not clear-cut. Hence, the categorization of noun referents into mass and count cannot be based completely on their conceptual-semantic characteristics. Indeed, the hypothesis of a conceptual-semantic distinction has sometimes even been described as being arbitrary or idiosyncratic (Bloomfield, 1933; Semenza et al., 1997; Gillon et al., 1999). Nevertheless, Wisniewski et al. (2003) suggested that the syntax of mass and count nouns is systematically related to the conceptual distinction in the mind of speakers. The interpretation of an aspect of reality (conceptualization) as an individual or non-individuated entity^3^ by a person or group of people determines the use of count or mass syntax. Within this *cognitive individuation hypothesis* it is assumed that how people perceive and interact with entities influences their categorization into mass or count. Wierzbicka (1988) believed that one of the important factors is the ease with which several elements of an entity can be distinguished. This assumption was supported by Middleton et al. (2004) who demonstrated that participants judged count noun aggregates (e.g., toothpicks, nuts, olives) as being easier to perceptually distinguish than mass noun aggregates (e.g., coal, popcorn, hair). Another important factor was considered to be the frequency with which people interact with individual elements or with multiple elements of aggregates. Regarding this hypothesis, Middleton et al. (2004) provided evidence that people interact more often with individual (one or a few) elements of count noun aggregates. However, people tend to interact more often with multiple (many) elements of mass noun aggregates. In other words, Middleton et al. proposed that the syntactic distinction is based not just on the semantics of those words (Bates and MacWhinney, 1982; Langacker, 1987) but also on the way people conceptualize these entities as being distinguishable and individual in their usage. Iwasaki et al. (2010) found further support for this theory by analysing substitution errors in the Japanese language. As noted above, Japanese speakers do not possess the grammatical distinction between mass and count nouns. However, the speakers were still found to be sensitive to conceptual distinctions related to English mass and count noun referents. This was shown by the fact that the majority of Japanese substitution errors shared the English mass/count status of the target word (e.g., target word: beer; substitution error: wine). Further support for a conceptual-semantic distinction between objects and substances has been found in several studies involving acquisition of novel names in children and adults (Soja et al., 1991; Imai and Gentner, 1997).

In sum, it appears that there is a broad conceptual-semantic difference between mass and count noun referents, which is to some extent reflected in the syntactic distinction. However, it has also been shown that conceptual-semantic and syntactic characteristics do not always correspond, hence, entities which can be counted and are easy to perceptually distinguish are not always count nouns (e.g., mass nouns: broccoli, asparagus) and substances or aggregates are not necessarily mass nouns (e.g., peas, lentils) (Vigliocco et al., 2002).

The contrast between mass and count nouns is also manifested in morphological and syntactic structures. One major difference between mass and count nouns is evident from the category name “countability”: count noun referents can be counted and count nouns can therefore be combined with numerals (e.g., English: **two** bicycles; French: **deux** vélos; Dutch: **twee** fietsen). Countability also implies that count nouns can be pluralized, which is mostly marked morphologically (e.g., English: bicycle vs. bicycle**s**; French: vélo vs. vélo**s**; Dutch: fiets vs. fiets**en**). Mass noun referents can generally not be counted and hence mass nouns are not combined with a numeral (e.g., English: ^*^two honey; French: ^*^deux miel; Dutch: ^*^twee honing) nor morphologically marked for plural (e.g., English: ^*^honeys; French: ^*^miels; Dutch: ^*^honings) (Semenza et al., 1997; Gillon et al., 1999; Wisniewski et al., 2003; Taler and Jarema, 2007). The count/mass status of a noun can also determine the form of a noun phrase. Count nouns can take an indefinite determiner (e.g., English: **a** bicycle; French: **un** vélo; Dutch: **een** fiets) while mass nouns can only take definite determiners (e.g., English: ^*^a honey; French ^*^un miel; Dutch ^*^een honing). Some languages also have some determiners which are specific to either mass or count [English: many_count_, few_count_; much_mass_, little_mass_; German: einige_count_ (several), etwas_mass_ (some)] or the same determiners but marked differently for countability (German: viele_count_, wenige_count_; viel_mass_, wenig_mass_). However, mass nouns mostly share certain determiners with singular (e.g., English: this honey/bicycle; French: ce miel/vélo; Dutch: deze honing/fiets) or plural count nouns (e.g., English: some honey/bicycles; French: beaucoup de (many) miel/vélos; Dutch: veel (many) honing/fietsen). Finally, the count/mass status of the subject in a sentence can determine the verb form. In order to form subject/verb agreement, in some languages (e.g., French, Dutch, German) the verb has to be conjugated for third person and/or plural morphology depending on whether the subject is a mass noun or a plural count noun [e.g., Le riz cuit (French)., De rijst kookt (Dutch)., Der Reis kocht (German) (The rice cooks.) vs., Les pommes de terre cuisent (French)., De aardappels koken. (Dutch), Die Kartoffeln kochen. (German) (The potatoes cook.)]. English is one of the many languages in which count nouns can be marked morphologically, syntactically or both. Plural count nouns are marked morphologically by the plural suffix *-s* which indicates clearly countability. Importantly, however, uninflected bare^4^ nouns are ambiguous in terms of countability: they can be singular count, or mass. The countability of nouns is marked syntactically within a noun phrase by a denumerator. Allan (1980) defined a denumerator as a quantifier which identifies one or more discrete entities and can be substituted for a natural number (e.g., one, two, no, all) within any noun phrase without changing the grammaticality of the noun phrase. The noun “chair,” for example, a count noun, can be combined with the denumerator “a” in a phrase such as “a chair.” “A” is a denumerator because it can be substituted for the number “one” (one chair). The noun “honey,” however, is not countable, and cannot be combined with a denumerator (^*^a honey, ^*^one honey). Allan considered mass nouns as morphologically and syntactically *unmarked* compared to count nouns due to the absence of denumerators or often of any determiner, in English^5^.

Languages such as English, Dutch and French, where countability is morphologically marked for number on nouns and constituents of a noun phrase and also by countability specific determiners, are referred to as “number marking languages” (e.g., Chierchia, 2010). However, Chierchia (2010) suggests that there are two other ways that languages can linguistically realize the mass/count distinction. Although the focus of this review is on mass and count nouns, we will briefly discuss these alternative linguistic realizations of the conceptual differences underlying the mass/count distinction (e.g., atomic, individuated).

First, in “nominal number neutral languages” (e.g., Dëne) nouns are not morphologically marked for number, instead countability is grammatically marked by numerals where only count nouns can be combined with numerals (similar to English, Chierchia, 2010). Second, Chierchia (2010) argues that countability is marked with classifiers in “classifier languages” (e.g., Mandarin Chinese, Korean). Classifiers are words or morphemes that can be considered to “classify” the noun dependent on the type of its referent. In classifier languages, nouns must be accompanied by classifiers in certain grammatical contexts. For example, when the noun is counted or specified, in other words, when it is combined with a demonstrative (e.g., that) or a numeral (e.g., three). In such languages, a phrase such as “three people” needs to be expressed as “three X people,” where X is the classifier appropriate to the noun for “people.” The classifier is specific to a noun or a group of noun referents and categorizes them based on the noun referents' physical, functional or social features. In classifier languages, such as Chinese Mandarin, nouns are not marked for number and, hence cannot be distinguished for countability. In the past, this has led to the view that all nouns in Mandarin Chinese are either mass nouns (e.g., Krifka, 1995; Chierchia, 1998) or that the mass/count distinction does not exist in this language (e.g., Cheng and Sybesma, 1998, 1999). However, Chierchia (2010) argues that the grammatical mass/count distinction is indeed represented, through classifiers which are specifically used for mass or count nouns. For example, “container classifiers” refer to all kinds of containers [e.g., “ebi” (glass)] and are combined with mass noun entities to define its quantity [“yi bei pijiu” (a glass of beer)]. “Individual classifiers” classify individual objects and therefore count noun entities [e.g., “tiao” which indicates “a long thing” and can therefore be combined with nouns such as “shengzi” (rope)] (see Gao and Malt, 2009). However, the relationship between classifier type and mass or count noun entities is not always transparent. For example, the classifier “kuai” (chunk) can be used with a mass noun entity to refer to “a piece of pork” and with count nouns which are chunky, such as rock (Liu, 2012). This example seems similar to determiners, such as “that” in English which can be combined with both mass and singular count nouns while countability is not grammatically marked by the determiner type or the noun's morphology.

In the next section, we will discuss the extent to which the different syntactic and morphological characteristics of mass and count nouns have been considered and explained in theories of language processing.

### Representation of countability in psycholinguistic theories of language comprehension and production

If the distinction between mass and count nouns is primarily syntactic, then the question emerges whether, for nouns, countability information is stored as a lexical-syntactic attribute, such as [count] and/or [mass] or whether the mass/count status of a noun is computed on the basis of the noun's semantic characteristics each time a noun is perceived or produced^6^. There has been remarkably little attention paid in the literature to this question, with only three explicit discussions, namely Taler and Jarema (2006); Barner and Snedeker (2005, 2006) and Levelt (1989). We will discuss these in turn. However, as questions regarding the representation and processing of mass and count nouns are similar to those concerning grammatical gender (e.g., masculine, feminine; see Schriefers and Jescheniak, 1999), we will refer to theories that discuss representation of grammatical gender where relevant.

**Figure 1:**
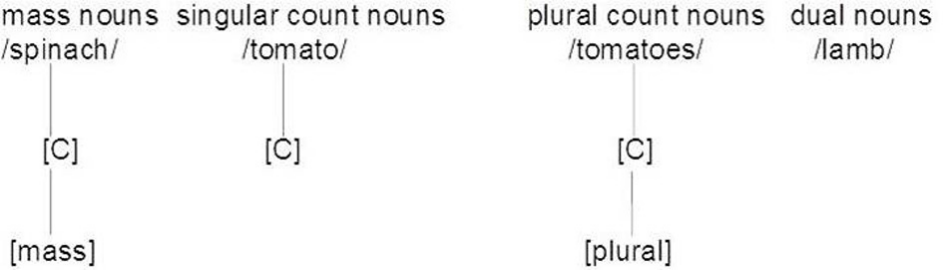
**Lexical representation of mass, count and dual nouns adapted from Taler and Jarema (2006, Figure 4, page 49)**.

Taler and Jarema (2006) argue that mass nouns, count nouns and dual nouns (nouns that can be both mass and count) are represented differently in the mental lexicon. According to their theory, nouns possess a node [countability] ([C]; see Figure 1). Noun categories differ in the specification of the [C] node. For mass nouns, the [C] node is further specified as mass [M], while count nouns only possess a bare [C] node. The bare [C] node is seen as the minimal structure which is necessary for nouns to form a valid representation. This account diverges from that of Allan (1980) who regarded mass nouns as the basic unmarked form.

**Figure 2:**
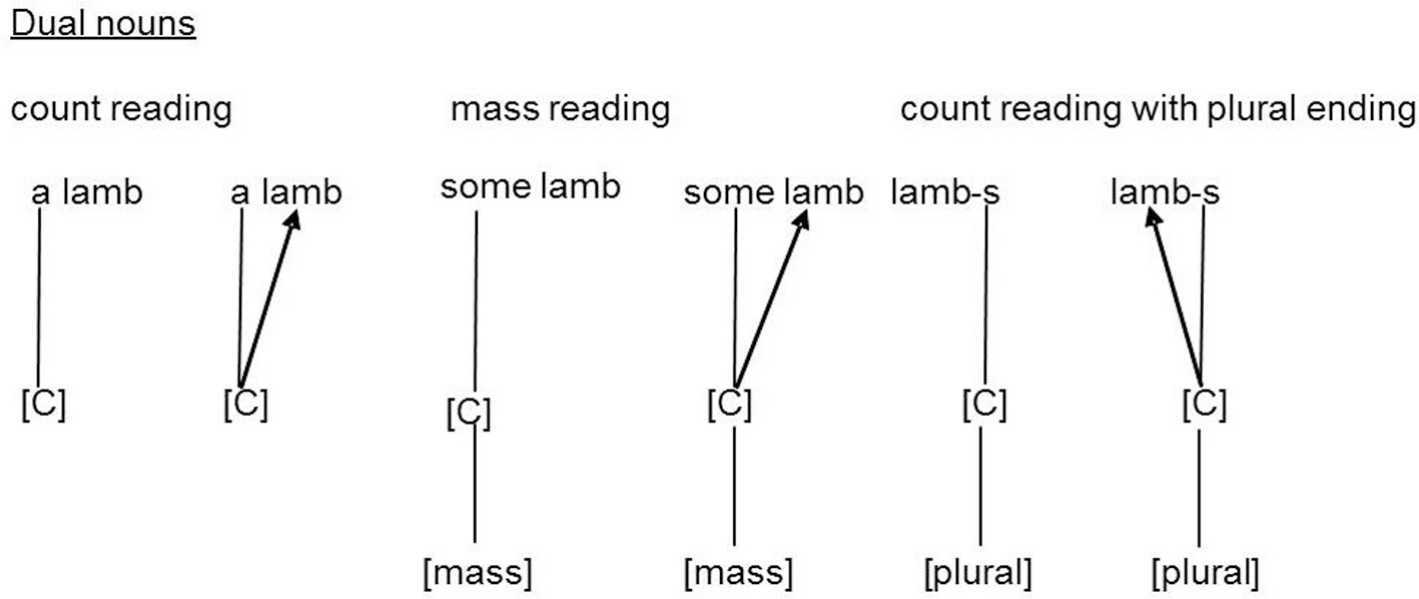
**Application of the lexical rule “countability by context” adapted from Taler and Jarema (2006, Figure 5, page, 50)**. Representation of the lexical-syntactic specification of dual nouns for countability by determiners or affixes during bare noun/noun phrase processing. The lexical-syntactic information [C] (count) or [mass] of the determiners “a”/“some” and the plural affix “–s” spreads automatically to the dual noun “lamb” (to be read from left-to-right). The application of the lexical rule on dual nouns in a grammatical context is regarded as an automatic and mandatory process (Taler and Jarema, 2006).

Taler and Jarema (2006) suggest further that dual nouns contrast with mass and count nouns by having no [C] node. To become valid, dual nouns require specification at the surface level, depending on the context, by means of a rule which Taler and Jarema (2006) named *countability by context* (CBC). Dual nouns can become specified by the determiner (e.g., a, much, many). Determiners also have countability nodes and the determiner is able to spread its countability node [C] to a dual noun representation. Dual nouns can be recognized as mass or count nouns through inheriting the countability attribute of the determiner (see Figure 2).

**Figure 3:**
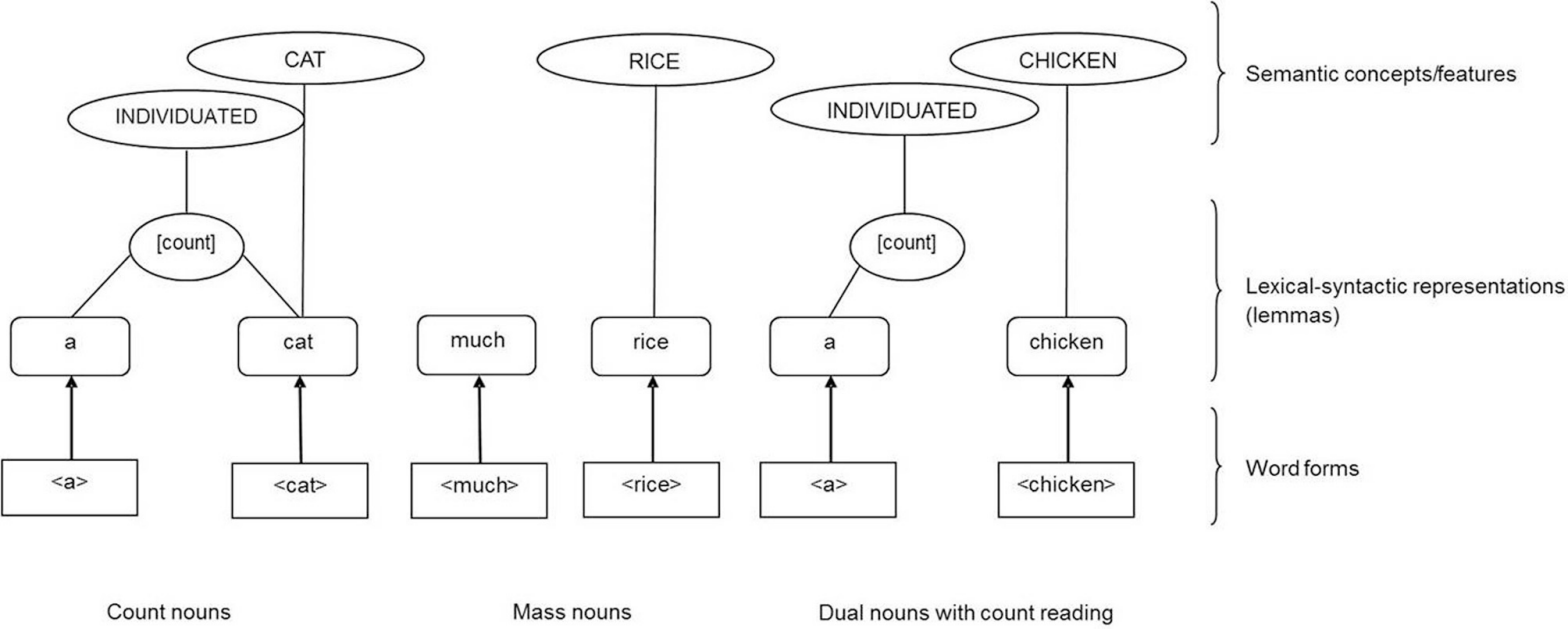
**Lexical representation of count, mass and dual nouns with count reading adapted from Barner and Snedeker (2005, 2006)**. The mass-count distinction is based on the single lexical-syntactic attribute [+ individual]. Count nouns and their syntactic context (e.g., count noun determiners: a, many) activate the lexical-syntactic attribute [+ individual] which allows the semantic and/or conceptual specification for “individuated.” To adapt Barner and Snedeker's theory to other theories (Levelt et al., 1999; Taler and Jarema, 2006), we use the term [count] for the attribute [+ individual]. Mass nouns and dual nouns as well as their syntactic context (e.g., mass noun determiners: much) lack the lexical-syntactic attribute [count]. Being unspecified for countability and individuation, mass nouns and dual nouns can inherit the lexical-syntactic and semantic specification of the syntactic context (e.g., the count noun determiner “a” assigns its [count] specification to the dual noun “chicken” and leads to a count reading of and a semantic interpretation of “chicken” as individuated entity).

Barner and Snedeker (2005, 2006) proposed a theory which contrasts with that of Taler and Jarema. Although they also propose that mass and count nouns differ in a single lexical-syntactic attribute, for Barner and Snedeker, *count nouns* are specified for countability whereas mass nouns and dual nouns lack any lexical-syntactic specification. The count specification “licenses” count nouns to be conceptual-semantically specified as individuated (individual) entities. The lack of lexical-syntactic specification for mass and dual nouns leads to more flexibility and allows both noun groups to individuate depending on the syntactic context. For example, if a mass or dual noun is preceded by a determiner or quantifier which is specified lexical-syntactically for being count (e.g., a, many), the lexical-syntactic specification can lead to a count reading of dual nouns and to a conceptual-semantic interpretation of mass and dual nouns as referring to individuated entities (e.g., a water, many ironies; see Figure 3).

While Taler and Jarema (2006) and Barner and Snedeker (2005, 2006) are among the few to have proposed potential theories of the representation of mass and count nouns, their accounts remain underspecified and are not embedded into a larger psycholinguistic theory. Hence, they are neither specified for how processing occurs (e.g., does every noun possess its own [mass] and/or [count] attribute or do all nouns share the same [mass] and/or [count] attribute node) nor at which level of processing the attribute nodes [mass] and/or [count] are represented. Therefore, we will first outline a more complete psycholinguistic theory (Levelt et al., 1999) and then consider whether Taler and Jarema and Barner and Snedeker's accounts of countability could be integrated into such a theory. It is possible that such an integrated theory might be able to interpret results from experiments with mass and count nouns in language perception and production in a clearer and more transparent manner.

Levelt et al. (1999) developed an influential theory of spoken word production. Although this theory does not explicitly mention countability, it is one of the few theories which makes clear assumptions about the representation and processing of lexical-syntactic attributes. We will first introduce the general organization and processing of the current version of this theory (Levelt et al., 1999). Following this, we will describe the lexical-syntactic representation of countability in Levelt's (1989) earlier version of this theory and employ this, together with the current assumption about the representation of grammatical gender (Levelt et al., 1999) to generate an expanded theory which includes the lexical-syntactic attribute countability. Finally, Levelt et al.'s (1999) theory of language production will be extended to incorporate processing of lexical-syntactic attributes in word comprehension.

Even though Levelt et al.'s theory makes precise claims regarding architecture and activation flow, we remain neutral in regard to certain aspects which are not critical to the representation of lexical-syntactic information (e.g., representations at the level of lexical concepts in form of holistic concepts (Levelt et al., 1999) vs. semantic features (e.g., Dell, 1986; Dell et al., 1997); unidirectional, serial activation flow (Levelt et al., 1999) vs. cascading activation flow (Caramazza, 1997; Caramazza and Miozzo, 1997, 1998) or restricted interaction between levels (Rapp and Goldrick, 2000; Goldrick and Rapp, 2002; see also Vigliocco and Hartsuiker, 2002; Nickels et al., 2014).

**Figure 4:**
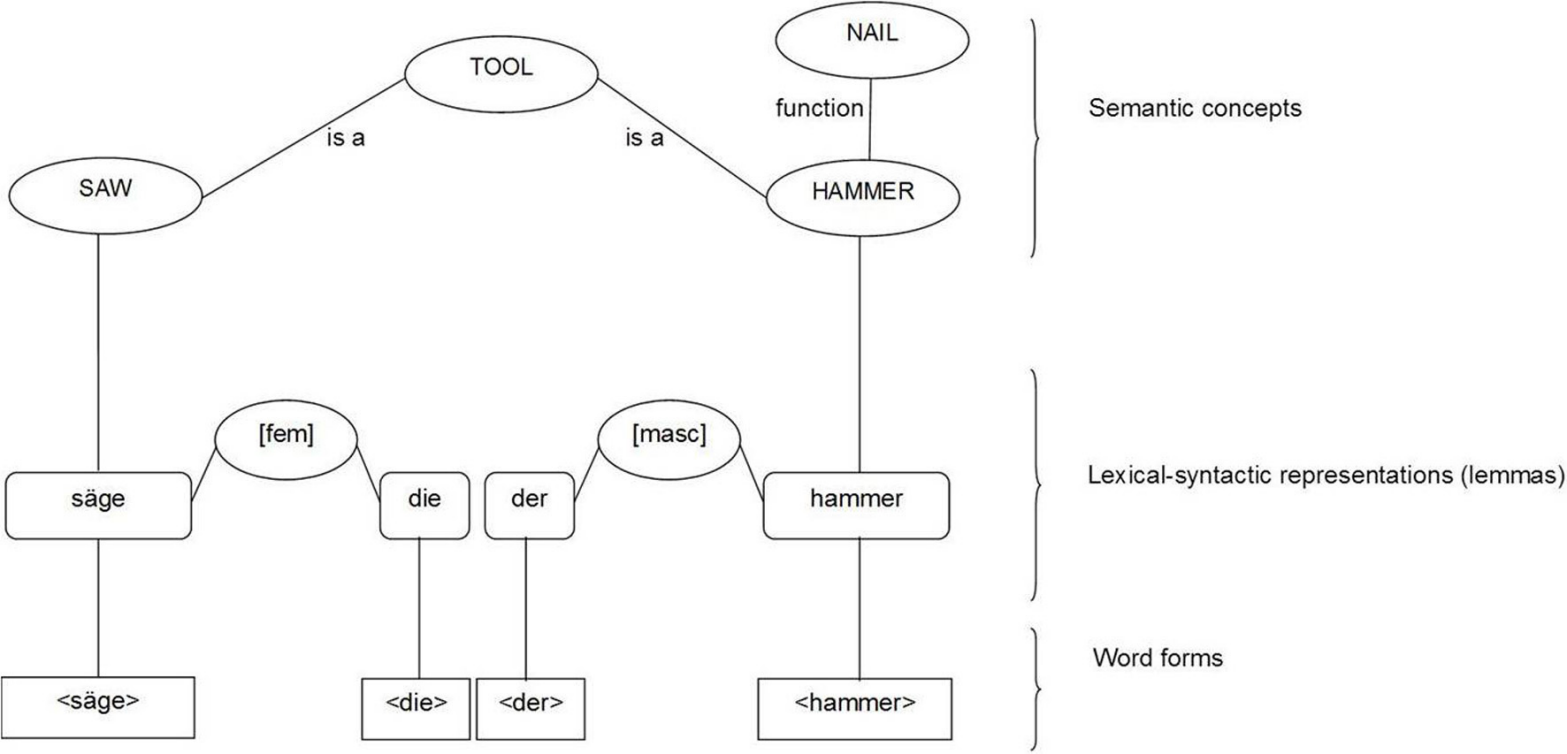
**Illustration of the different representations of the German nouns “Hammer” (hammer) and “Säge” (saw) at each level in Levelt et al.'s (1999) theory**. For the sake of clarity not all links are shown.

Levelt et al.'s (1999) theory incorporates five levels of linguistic processing: a level of lexical concepts (conceptual-semantic level), a lexical-syntactic (lemma) level, a word form level, a phonetic level and an articulatory level. The production of a meaningful word implies the activation of a concept. Concepts and the relationships among them are represented in form of nodes and the connections between the nodes (see Figure 4). Each conceptual-semantic node is connected with one lemma node and spreads activation to it. The highest activated lemma is selected.

Lemma nodes are *empty nodes*, which mediate between conceptual-semantic, lexical-syntactic and phonological information. Each lemma node is linked to lexical-syntactic attributes. Levelt et al. (1999) distinguish between two kinds of lexical-syntactic attributes: lexical-syntactic properties and lexical-syntactic features. Lexical-syntactic properties are fixed intrinsic attributes of a lemma (e.g., grammatical gender^7^). Lexical-syntactic features are variable extrinsic attributes which are set depending on the context or intention of the speaker (e.g., number: singular vs. plural). For clarity from here on, we will use the more explicit terms: “fixed intrinsic lexical-syntactic properties” to refer to lexical-syntactic properties, “variable extrinsic lexical-syntactic features” to refer to lexical-syntactic features, we will use the term “lexical-syntactic attributes” to refer to both features and properties. All lemmas with a given lexical-syntactic attribute are connected to the same abstract node which marks this attribute (e.g., there is a single node for the grammatical gender [masculine]) (Schriefers and Jescheniak, 1999). The lexical-syntactic nodes (e.g., [masculine],) are in turn connected to grammatically congruent lemma nodes, such as determiners and quantifiers (e.g., the German determiner “der_masculine_”). Consequently, the selection of a lexical-syntactic attribute affects grammatical encoding. For example, in German, the grammatical gender of a noun influences the choice of determiner [e.g., “**der_masc_** neue Hammer_masc_” (the new hammer)] and/or the suffix of the adjective [e.g., “neue**r_masc_** Hammer_masc_” (new hammer)]. Even though lexical-syntactic attributes are always activated when bare nouns, noun phrases or sentences are processed, they are only selected if they are grammatically required, such as for the selection of grammatically congruent determiners (see also Schriefers et al., 2002).

Fixed intrinsic lexical-syntactic properties, such as grammatical gender are selected through activation from the noun lemma, which flows unidirectionally to the property node and further to grammatically congruent lemma nodes (e.g., determiners). In contrast, variable extrinsic lexical-syntactic features, such as number are predominantly, or even exclusively, activated and selected via semantic concepts/features in language production. For example, the lexical-syntactic feature [plural] is activated via the semantic concept/feature MULTIPLE. We will address below whether countability might be considered as a fixed intrinsic lexical-syntactic property.

The selection of a lemma is the first stage of lexicalization. It is followed by the retrieval of the appropriate word form at the word form level (see Figure 4). Finally, selected word forms are phonetically and articulatory encoded at post-lexical levels in order to be converted into speech (Levelt et al., 1999).

How can countability be represented at the lexical-syntactic level within Levelt et al.'s (1999) theory? While Levelt et al. (1999) do not address this explicitly, Levelt (1989) described the differences between mass and count nouns briefly in his previous version of the theory. In addition, we can deduce further assumptions from the representation of other lexical-syntactic attributes, like grammatical gender, which Levelt et al. (1999) have explicitly addressed.

**Figure 5:**
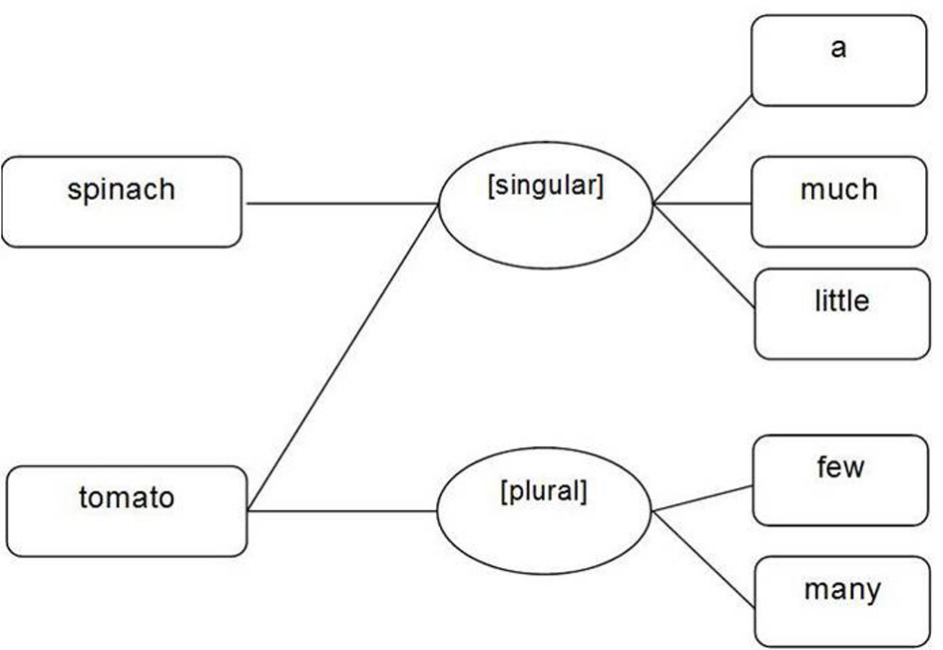
**Representation of mass nouns (e.g., spinach) and count nouns (e.g., tomato) at the lexical-syntactic (lemma) level according to Levelt (1989)**.

Unlike Taler and Jarema (2006) and Barner and Snedeker (2005, 2006), Levelt (1989) did not propose countability specific attributes, such as [mass] and/or [count] to distinguish between mass and count nouns. Instead, he postulated that the underlying difference lies in the number feature(s) to which a noun is connected. Count nouns, which can occur as singular and plural, are connected to the variable extrinsic lexical-syntactic features [singular] and [plural]. Mass nouns however, are linked to the single, and therefore fixed, lexical-syntactic attribute [singular]^8^ (see Figure 5).

However, Levelt did not consider that mass nouns can require different determiners and quantifiers to singular count nouns (e.g., much, little, some vs. a, one), a fact which cannot be explained by countability being represented via number attributes: both mass and count nouns would be connected to the same lexical-syntactic node [singular]. Although this lexical-syntactic node can be either fixed (for mass nouns) or variable (for count nouns), it cannot differ in the connections to the grammatically congruent determiner/quantifier lemma nodes. Consequently, the abstract node [singular] would be connected to determiner lemma nodes for singular count nouns (e.g., the, a) as well as with determiner lemma nodes for mass nouns (e.g., much, some, enough) which may also be associated with a plural meaning. Levelt's (1989) proposal of countability representation could theoretically lead to the selection of countability/number incongruent determiners for mass and singular count nouns (e.g., “a” for mass nouns, “much” for singular count nouns) and hence to the production of countability incongruent noun phrases and sentences (e.g., ^*^a rice, ^*^much car). This, however, is inconsistent with speech error data which shows that substitution errors of language unimpaired speakers are generally lexical-syntactically congruent with the target word (e.g., Berg, 1992; Del Viso et al., Unpublished manuscript). In sum, Levelt's (1989) proposal for the lexical-syntactic specification of mass and count nouns seems to be insufficient. We propose therefore an account which is based on the representation of the fixed intrinsic lexical-syntactic property gender in the more recent version of this theory (Levelt et al., 1999).

**Figure 6:**
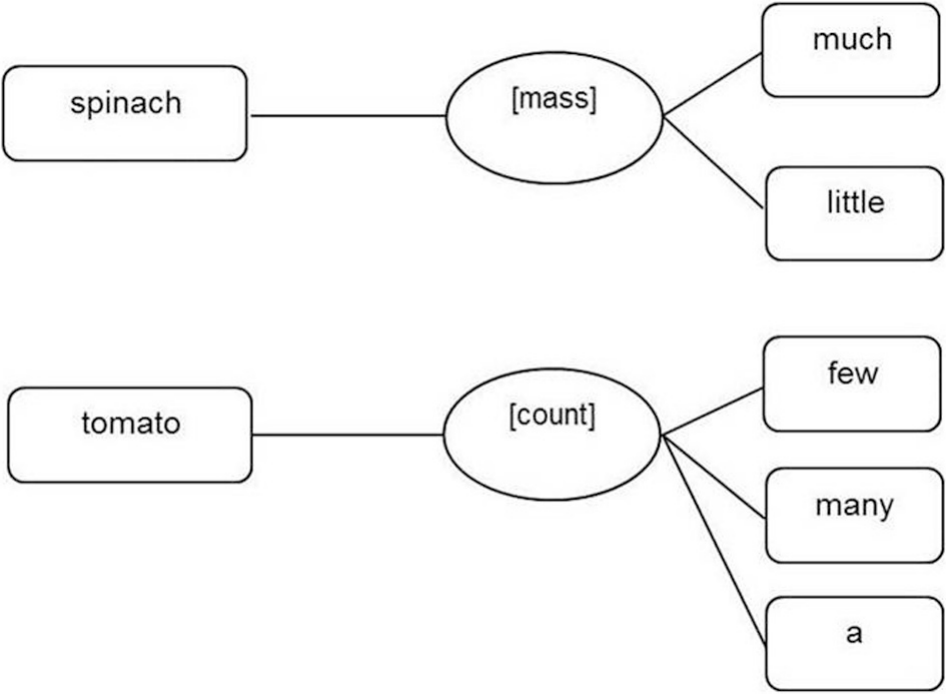
**Representation of mass nouns (e.g., spinach) and count nouns (e.g., tomato) at the lexical-syntactic (lemma) level derived from assumptions about the representation of the fixed intrinsic lexical-syntactic property gender in Jescheniak and Levelt (1994) and Levelt et al. (1999)**.

The lack of complete conceptual-semantic transparency of mass and count nouns within and across languages makes it unlikely that countability is represented in the form of extrinsic, variable, lexical-syntactic features like number. Instead, it seems more plausible that nouns are specified for countability in form of fixed intrinsic lexical-syntactic properties in a similar way to grammatical gender [supported by data from Steinhauer et al. (2001)]. Assuming that nouns are specified through fixed intrinsic lexical-syntactic [mass] and/or [count] properties, three forms of representation are possible. Mass and count nouns could be equally well specified with count noun lemmas being linked to a [count] property node and mass noun lemmas being linked to an independent [mass] property node at the lexical-syntactic (lemma) level (similar to the assumption for the representation of grammatical gender, see Figure 6).

**Figure 7:**
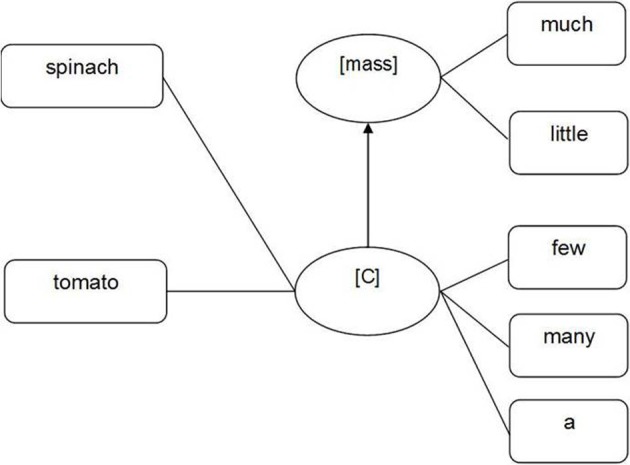
**Representation of mass nouns (e.g., spinach) and count nouns (e.g., tomato) at the lexical-syntactic (lemma) level derived from Taler and Jarema's assumption (2006)**.

**Figure 8:**
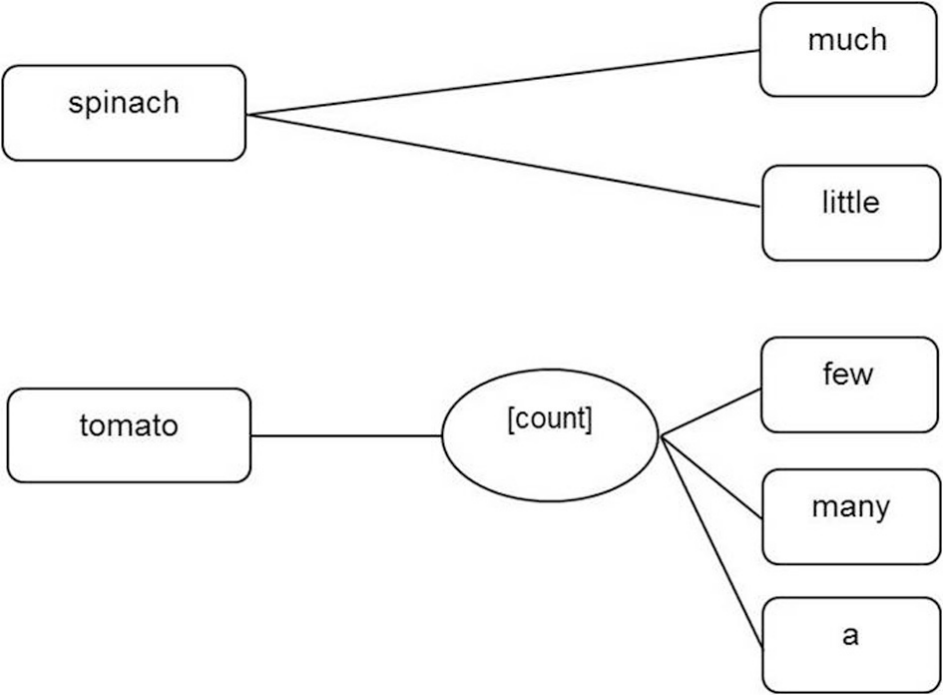
**Representation of mass nouns (e.g., spinach) and count nouns (e.g., tomato) at the lemma level derived from Barner and Snedeker's assumption (2005, 2006)**.

Another theory is that of Taler and Jarema (2006), discussed above. According to their theory both count nouns and mass nouns are linked to a countability property ([C]) which can be regarded as the unmarked or default property (see Figure 7). Mass nouns are further specified through a mass property—a marked property (see also Mondini et al., 2009). Alternatively, as described earlier in Barner and Snedeker's (2005, 2006) theory the specification of countability for count nouns could be implemented in the form of a [count] property at the lexical-syntactic level. The [count] property could be linked to a semantic feature INDIVIDUATED at the conceptual-semantic level. Mass nouns and dual nouns would remain syntactically unspecified for countability and semantically unspecified for individuation (see Figure 8).

**Figure 9:**
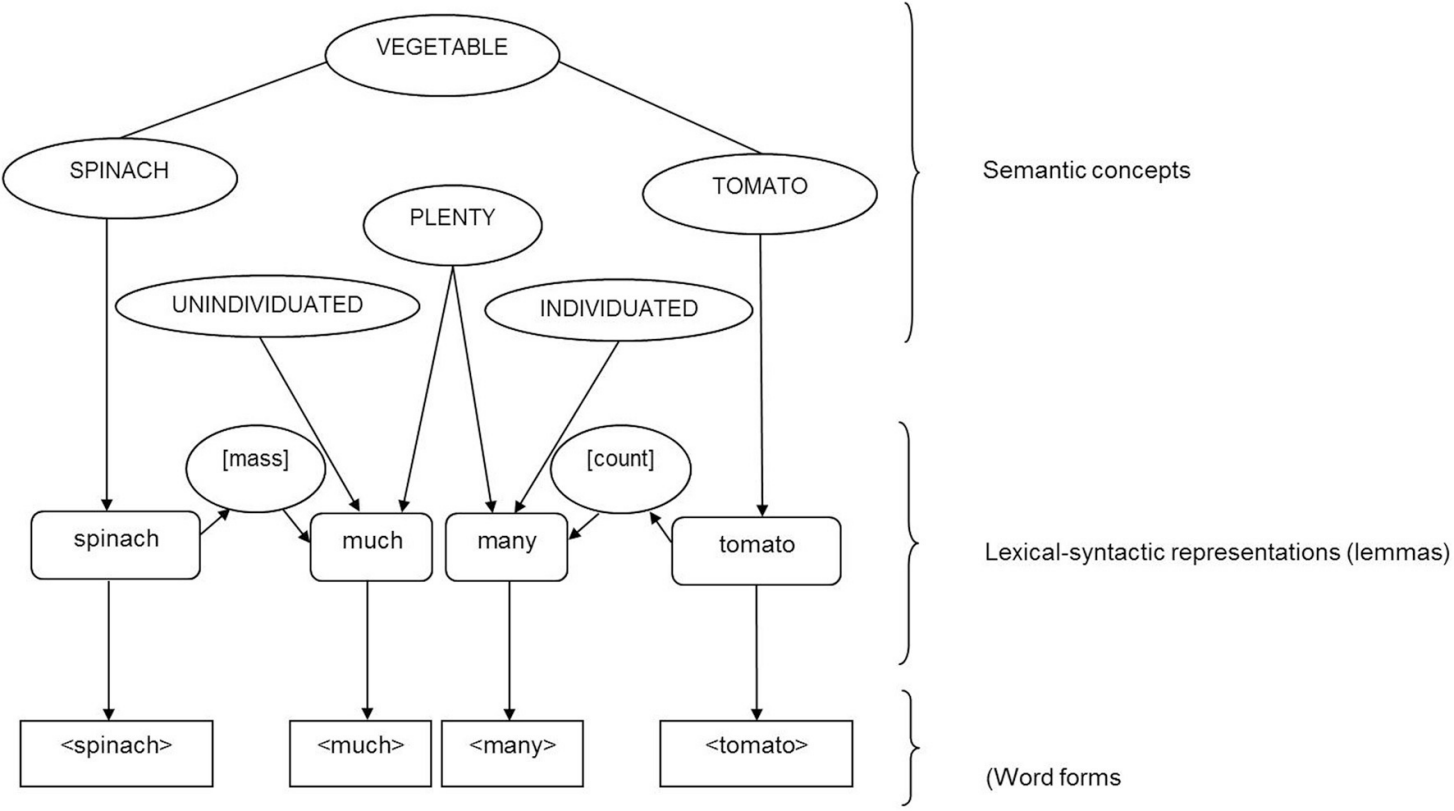
**Illustration of the processing and representation of mass nouns (e.g., spinach) and count nouns (e.g., tomato) in a theory of language production derived from Levelt et al.'s (1999) theory, assuming separate count and mass properties at the lexical-syntactic (lemma) level, and the semantic concepts/features INDIVIDUATED for count nouns and UNINDIVIDUATED for mass nouns at the conceptual-semantic level**.

Like grammatical gender, the countability of a noun can be regarded as a fixed intrinsic lexical-syntactic property which means it is predetermined for each noun lemma and cannot be influenced by context. In all three accounts above, an activated and selected noun lemma would spread activation to its [mass]/[count] property. Like other fixed intrinsic lexical-syntactic properties in Levelt et al.'s (1999) theory, [mass]/[count] properties would only become selected if they are required for grammatical computation, for instance to select a countability congruent determiner/quantifier (e.g., “much” for mass nouns vs. “many” for count nouns, see Figure 9) but not for the production of bare nouns (see also Roelofs, 1992, 1993; Schriefers and Jescheniak, 1999). However, Levelt et al. do not specify the precise mechanism by which a lemma “knows” whether or not grammatical information should be selected dependent on the context. Presumably there must be, minimally, an interaction with the sentence level. It is also possible that quantifiers, like “much” or “many,” have additional semantic feature/concept representations, such as PLENTY and ATOMIC/INDIVIDUATED or NONATOMIC/UNINDIVIDUATED. The target determiner lemma node could be selected through activation of the lexical-syntactic property [mass]/[count] and the conceptual-semantic representation.

We noted in the section above that some nouns are “dual” nouns with both mass and count interpretations (e.g., lamb, fish). While we primarily concentrate on those nouns which are not dual nouns, we will briefly consider how these dual nouns might be represented. Probably the most straightforward account is that these nouns are a special case of homophones—they have the same word form but different meanings, and different lexical-syntactic properties (i.e., [mass] vs. [count]). This account, unlike that of Taler and Jarema (2006) avoids the need to suggest a different lexical-syntactic representation for dual nouns to other (non-dual) nouns.

**Figure 10:**
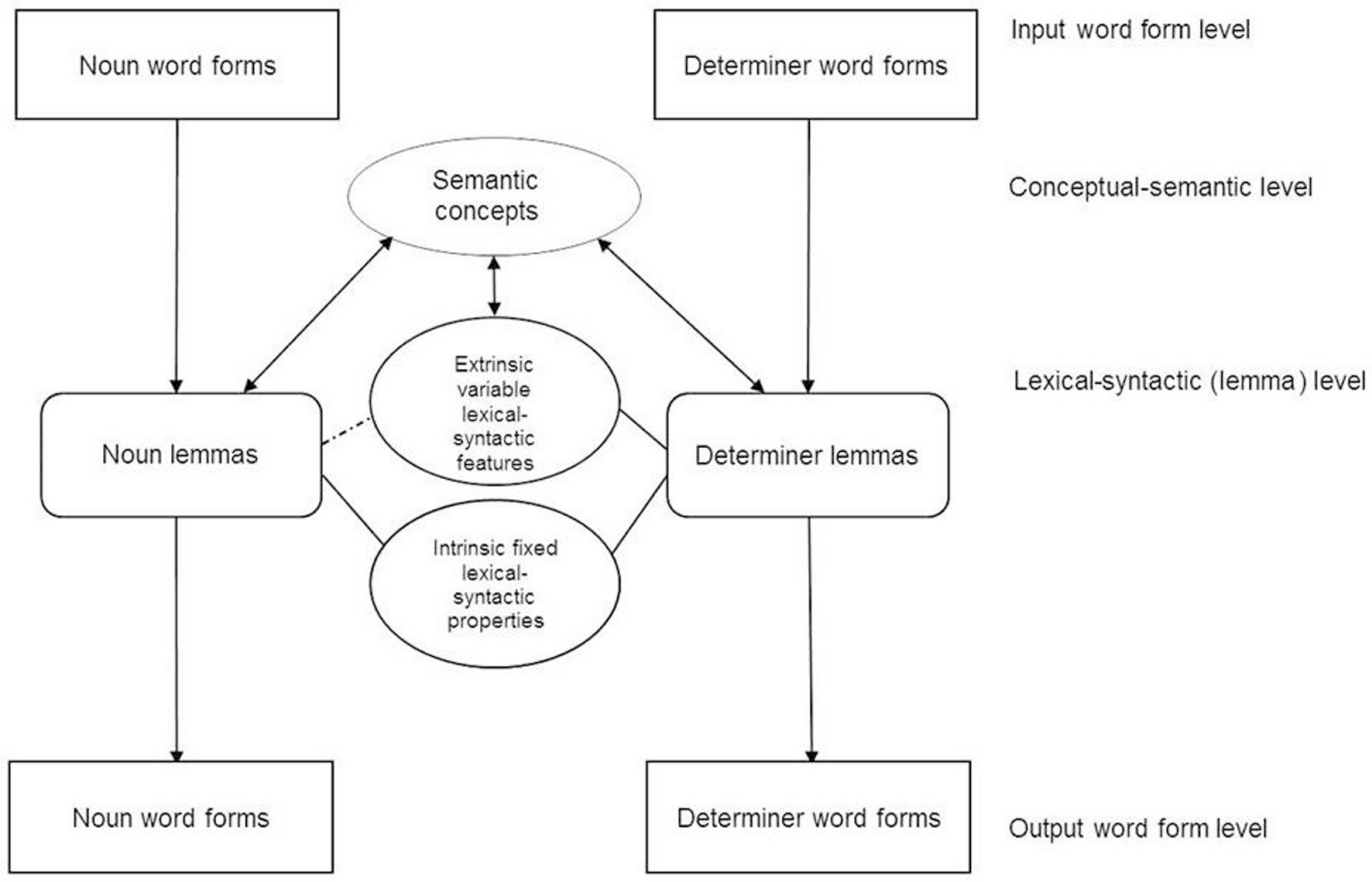
**General illustration of the different levels involved in language processing and production of noun phrases derived from Levelt et al.'s (1999) theory**. Extrinsic variable lexical-syntactic features are activated exclusively or at least predominantly through semantic features/concepts hence the dotted link between noun lemma and features.

Having extended Levelt et al.'s (1999) theory of language production to countability, the question remains regarding how mass and count nouns might be represented and processed in language comprehension? Levelt et al.'s (1999) theory of spoken word production was developed further by Roelofs (2003) who described word comprehension and its relationship to spoken word planning within Levelt et al.'s (1999) theory. The comprehensive description of the lexical-syntactic (lemma) level makes Levelt et al.'s theory attractive for an extension to the process of language comprehension. Such an extended theory is required in order to be able to account for effects of countability in, for example, semantic categorization. Two levels of the word production model can be directly assigned and incorporated to a model for word comprehension: the conceptual-semantic and the lexical-syntactic (lemma) level (see Figure 10). Levelt et al. (1999) regard both levels as modality-neutral and therefore accessible for language production and comprehension. In order to comprehend a noun, auditory or written input would activate the corresponding input representation(s) at the modality-specific (phonological or orthographic) word form level. The lemma node forwards activation to its lexical-syntactic attributes and the associated semantic concept.

In sum, we have extended Levelt et al.'s (1999) theory of language production to include language comprehension processes and a specification of the representation of countability at the lexical-syntactic (lemma) level. However, there remain three potential variants of this extended theory. The first includes both mass and count properties, we will refer to this as the *Count And Mass Marked hypothesis* (see Figure 6, earlier). The second variant of the theory has only mass nouns marked for countability, with count nouns unmarked, we will refer to this as the *Count Unspecified Mass Marked hypothesis* (see Figure 7, earlier). The third variant of the theory has only count nouns marked for countability, with mass nouns unmarked, we will refer to this as the *Mass unspecified Count Marked hypothesis* (see Figure 8, earlier).

To develop, test and extend theories and distinguish between competing theories, researchers rely on experimental data. In the next section we will give an overview of experimental studies which have investigated processing of mass and count nouns in language production and comprehension. Following this, we will discuss the interpretation of these results within the different theoretical accounts.

First, we will introduce language production studies (Vigliocco et al., 1999; Biedermann et al., 2008) which assessed whether lexical-syntactic information like countability can be accessed during a ToT state, when a person has access to the semantics of a word but cannot retrieve the word form itself. Subsequently, we will discuss studies which investigated processing of mass and count nouns in language comprehension (e.g., Gillon et al., 1999; Mondini et al., 2009). Experimental investigations of mass and count nouns have also been carried out with individuals with language impairments (e.g., Semenza et al., 1997; Vigliocco et al., 1999; Herbert and Best, 2010). In the last section, we will present those case studies which demonstrate selective impairments of mass nouns.

## Investigations of mass and count nouns in language production: availability of mass/count information in tip-of-the-tongue state

Vigliocco et al. (1999) examined the availability of mass and count information during a TOT state. The TOT state is a common phenomenon which is experienced by speakers of any language. Speakers in a TOT state feel that they know the target word without being able to retrieve and produce the word form at that particular moment. Nevertheless, they might be able to retrieve pieces of phonological and/or grammatical information (e.g., initial phoneme, the number of syllables, the grammatical gender of a noun). While TOTs occur spontaneously, they can also be induced experimentally by giving a person a definition or picture of a low-frequency word. In Vigliocco et al.'s (1999) experiment, native English speakers were tested using mass and count nouns of low frequency. The participants were asked to name a noun when provided with a definition which was read aloud by the examiner. When participants could not produce the target, they were asked to answer a questionnaire, composed of three different sections. In the first section, participants were asked to choose the correct context for the word: There is __/There is a __.; There won't be much__/There won't be many__.; There is some__/There are a few__. These questions probed the availability of lexical-syntactic information regarding mass/count status. In the second part, the participants' task was to guess the number of syllables in the word. In the final step, they were required to guess any letters or sounds and their positions within the word. The questions in sections two and three probed the accessibility of metrical and segmental information independent of the retrieval of the word form. The examiner then provided the participants with the target. The response was scored as a positive TOT state if the target word matched the word which the participant had in mind and as negative TOT state (i.e., not in a TOT state) if both words did not match. The comparison of positive TOT states and negative TOT states revealed that lexical-syntactic information was significantly more accessible when participants were in a (positive) TOT state than when they were not.

Vigliocco et al. (1999) found similar results for an anomic aphasic individual, MS who suffered from severe naming difficulties while his semantic, lexical-syntactic and post-lexical processing remained unimpaired. Similarly to language unimpaired individuals, MS was able to access lexical-syntactic mass/count information when the lexical retrieval of the phonological word form failed.

Vigliocco et al. concluded that lexical-syntactic attributes (i.e., countability) can be retrieved independently of the word form. Further, tests of independence showed no correlation between the retrieval of phonological and lexical-syntactic information. Based on these results, Vigliocco et al. concluded that word form retrieval is independent from lexical-syntactic information. The results were replicated by Biedermann et al. (2008) with language unimpaired speakers for English and extended to German.

In summary, results of both TOT studies give evidence for a lexical-syntactic representation of countability information. Moreover, failure to access lexical-syntactic mass/count information even with semantic access (assured through provision of definitions), supports the argument that the mass/count status of words cannot be fully derived from their semantics. Hence, the results support the proposal that in Levelt et al.'s (1999) theory countability is represented in form of fixed intrinsic lexical-syntactic properties at the lexical-syntactic (lemma) level. Moreover, these experiments show that countability information at the lexical-syntactic level is separate from conceptual-semantic and from phonological information. However, the fact that independent access of phonological information from lexical-syntactic information was found requires the possibility that (at least partial) word form access can be achieved without selection of lexical-syntactic attributes (Schriefers et al., 2002; Biedermann et al., 2008). The results from the two TOT studies do not allow us to draw further conclusions about lexical-syntactic markedness and therefore to distinguish between the three hypotheses discussed earlier.

## Investigations of mass and count nouns in language perception/comprehension

In this section we focus on studies which have investigated lexical-syntactic and/or conceptual-semantic differences in processing of mass and count nouns. We limited our investigations to those studies which exerted sufficient experimental control and to lexical-syntactic studies which used phrases/sentences.

Steinhauer et al. (2001) looked at syntactic and semantic processing of mass and count nouns in sentences in an electrophysiological (EEG) study. Participants were asked to read semantically plausible and implausible sentences which contained either a mass or a count noun and had to judge a sentence's acceptability by button press (yes or no buttons). The ERP results revealed a grammatically related frontal negativity effect during reading of semantically plausible sentences with mass/count nouns. The grammatical mass/count effect was unrelated to posterior semantic effects (N400) which were found in semantically implausible sentences. The grammatical effect found for mass and count noun processing provides evidence for a syntactic rather than a semantically based mass/count distinction.

Gillon et al. (1999) investigated mass and count nouns in lexical decision involving morphosyntactic priming. The test material comprised grammatical and ungrammatical prime-target combinations which consisted of a determiner or adjective and a mass noun (non-atomic mass nouns: water; atomic mass nouns: furniture) or count noun stimulus (e.g., grammatical prime: “much_mass_,” ungrammatical prime: “^*^many_count_,” target for lexical decision: MUD_mass_). The primes were presented prior to the target. The results revealed an interaction with condition: atomic mass nouns showed shorter reaction times in the ungrammatical condition than in the grammatical condition whereas the opposite pattern was found for count nouns and non-atomic mass nouns which showed shorter reaction times in the grammatical condition than in ungrammatical combinations. The longer reaction times for count nouns and non-atomic mass nouns in the ungrammatical condition were accounted for by a mismatch between the attributes which are activated by the prime determiner and target noun (e.g., “much” activates the lexical-syntactic attribute [mass] and the target noun activates the count reading). The shorter reaction times for atomic mass nouns in the ungrammatical condition were explained by semantic priming of the semantic feature ATOMIC which is shared by count nouns and count noun determiners but also by atomic mass nouns.

Processing of mass and count nouns (abstract and concrete) was tested further by El Yagoubi et al. (2006) in a grammaticality judgment task in which participants were asked to judge sentences with mass or count noun syntax for grammaticality. Results showed that participants needed significantly longer decision times for grammatically correct sentences with concrete mass nouns compared to the other types of sentences.

Bisiacchi et al. (2005) and El Yagoubi et al. (2006) investigated conceptual-semantic processing of concrete mass and count nouns in a semantic categorization task. Prior to Bisiacchi et al.'s experiment, participants were instructed about the semantic differences between mass and count nouns. During the task, participants were required to categories visually presented words into mass and count by button press. The results showed that participants required longer processing times for the categorization of mass compared to count nouns (see also Mondini et al., 2008). The ERP results showed further a significant difference in early automatic (N150) activation: Activation patterns for mass nouns were more widespread including the right hemisphere (see also Mondini et al., 2008). In the task by El Yagoubi et al., participants were asked to categories mass and count nouns into the categories abstract or concrete by button press. The results revealed a significant interaction with abstract count nouns requiring longer processing times than any other noun category.

In summary, semantic categorization tasks (Bisiacchi et al., 2005; Mondini et al., 2008) have shown countability specific effects, interpreted as reflecting a semantic difference between mass and count nouns, where mass nouns represent typically substances or non-atomic entities and count nouns concrete atomic objects. Moreover, concrete (atomic) mass nouns which are semantically more similar to count nouns have been shown to be processed differently to other (non-atomic, abstract) mass nouns (e.g., substances) (Gillon et al., 1999; El Yagoubi et al., 2006). Similarly, semantic categorization of abstract count nouns was more difficult than of mass nouns and concrete count nouns (El Yagoubi et al., 2006). This suggests that mass nouns with semantic characteristics that are atypical of the category in general (e.g., atomic mass nouns) are harder to process. Taken together, the results suggest that both mass and count nouns are semantically specified for countability and therefore do not support fully Taler and Jarema (2007) or Barner and Snedeker's (2005, 2006) theory where either mass or count nouns, but not both, are semantically specified for UNINDIVIDUATED / INDIVIDUATED. We will revisit the finding that the semantic representations of mass nouns can lead to longer processing times compared to count nouns (Bisiacchi et al., 2005; Mondini et al., 2008) in the discussion.

## Investigations of mass and count nouns through case studies of individuals with language impairments

We now turn to explore individuals with language impairment to investigate the representation of countability. Some individuals with language impairments have been shown to process mass and count nouns similarly to participants without language impairment. For example, Taler and Jarema (2006) looked at individuals with Alzheimer's disease and mild cognitive impairments and found no specific deficits in processing of bare mass and count nouns in a lexical decision task. Taler et al. (2005) and Garrard et al. (2004) found similar results in a study with two individuals with semantic dementia, JH and Oscar. However, there have also been a number of reports of specific impairments in the processing of mass and count nouns which we describe in detail below.

Semenza et al. (1997) reported the case of a 73 years old, Italian speaking woman, FA, who had anomic aphasia and showed difficulties with mass noun grammar. Her performance on mass and count nouns was investigated in seven tasks (i.e., two naming tasks; two semantic tasks; three morphosyntactic tasks). FA did not show a countability specific effect in the first four tasks: naming to definition (e.g., What animal barks?), naming through sentence completion (e.g., That… is chained because otherwise it would bite.), semantic judgments (judging the acceptability of written sentences; e.g., The dog mews.), semantic association (matching of written words which are semantically associated; e.g., “dog” to either “bone” or “flower”). However, she showed an isolated impairment of mass nouns in the last three tasks which focused on lexical-syntax. In the first task, FA was asked to judge the grammaticality of sentences which involved correct or incorrect mass/count noun determiners and quantifiers. (e.g., ^*^There is *much* desk in this classroom.). In another task she was required to complete sentences by choosing the correct determiner or quantifier (e.g., I would like… water, please. ^*^a, some, ^*^many). In the final task she was asked to form a semantically and syntactically correct sentence with a target noun (count or mass) and a semantically associated noun (e.g., roll/butter). Overall, FA's errors resulted from either treating mass nouns as count nouns by pluralizing them and choosing count noun determiners and quantifiers, or by substituting and omitting the mass nouns. She showed no consistency in the affected items and in the type of errors she made. Semenza et al. ascribed her deficit to an isolated problem with the grammar of mass nouns due to a loss or inaccessibility of their grammatical rules.

In a second single case study, Semenza et al. (2000) described CN, a 72 years old woman with anomic aphasia who showed a pattern of performance opposite to that of FA. CN's performance on mass and count nouns was investigated with six of the tasks which were used in Semenza et al's (1997) study. The tasks were repeated twice, 2 and 3 months later. CN's performance in the syntactic and semantic tasks was no different to that of the control group. However, the name retrieval tasks revealed deficits particularly with regard to count nouns. In the later assessments, CN's performance in naming remained impaired, but again with better performance for mass nouns. Semenza et al. (2000) proposed an impairment in the lexical retrieval of count noun word forms.

How far does the data from Semenza and colleagues, inform our understanding of the representation of countability? FA had impairments in morphosyntactic tasks that were restricted to mass nouns, and Semenza et al. (1997) ascribed her deficit to a loss or inaccessibility of grammatical rules for mass nouns. In terms of our proposed extension of Levelt et al.'s theory, FA's difficulties can be described as damage to the lexical-syntactic [mass] node at the lemma level (under either the Count And Mass Marked or the Count Unspecified Mass Marked hypothesis). This would affect any task which required selection of the lexical-syntactic property [mass], but would leave processing of count nouns unaffected. FA's ability to name (bare) mass nouns supports Levelt et al.'s assumption that fixed intrinsic lexical-syntactic properties (e.g., grammatical gender, countability) are only selected when they are grammatically required. This assumption also predicts FA's intact performance for mass and count nouns in semantic tasks. Barner and Snedeker's theory (implemented as Mass unspecified Count Marked hypothesis) however, cannot explain a lexical-syntactic deficit restricted to mass nouns since mass nouns remain lexical-syntactically unspecified and hence cannot be selectively impaired at this level.

CN (Semenza et al., 2000) showed a deficit in naming bare count nouns but not mass nouns, while her performance on syntactic and semantic tasks remained unimpaired. This pattern of performance is difficult to interpret with an impairment at the lexical-syntactic level in any of the possible extensions of Levelt et al.'s theory described above. A count noun naming deficit would seem to imply damage to the [count] property at the lexical-syntactic level. However, since the production of bare nouns does not require selection of these properties, this cannot account for CN's impairment in naming count nouns. Nor would an impairment of the lexical-syntactic property [count] explain CN's intact performance on syntactic tasks since selection of lexical-syntactic mass/count information is required to retrieve the appropriate determiner/quantifier. One possibility is that CN's difficulties in naming count nouns originate at the conceptual-semantic level. As discussed earlier Barner and Snedeker (2005, 2006) and Gillon et al. (1999) suggest that mass and count nouns differ in their semantic features. Perhaps, then, CN has an impairment of count specific semantic features (e.g., INDIVIDUATED, ATOMIC). As naming relies on semantic information, damage of a core feature or features could affect naming performance of a whole category (Hillis and Caramazza, 1991). This account suggests that while semantic information is not sufficient to determine the lexical-syntactic mass/count status (as shown by the TOT studies described earlier), without the relevant semantic feature(s), noun representations cannot be accessed at the lexical-syntactic and/or word form level.

Another single case who showed an advantage for naming count nouns over mass nouns is reported by Herbert and Best (2010). MH was diagnosed with a non-fluent agrammatic aphasia and severe anomia. Her word reading was impaired due to deep dyslexia. MH's performance on tasks which demanded semantic and phonological processing and visual perception was in normal range. To investigate MH's processing of mass and count nouns, Herbert and Best conducted four different tests: (a) spoken picture naming of bare mass and count nouns, (b) syntactic judgment of determiner plus noun combinations, (c) repetition and reading aloud of determiner plus mass/count noun combinations, and (d) spoken picture naming with and without syntactic cues. MH showed particular problems in naming pictures of mass nouns compared to count nouns. Results of the cued picture naming task showed that MH's mass noun production improved when syntactic determiner cues were presented (“This is a/an…” for count nouns and “This is some…” for mass nouns). While naming of count nouns remained the same, the improvement in mass noun naming led to similar naming accuracies between mass and count nouns. In the syntactic judgment task, MH was presented with the picture of a count/mass noun and the two determiners “a” and “some” in spoken and written form. She was asked to decide which of the determiners could be combined with the name of the picture. Her results showed a preference for the determiner “a”/ “an” over “some.” Tests of repetition and reading aloud of noun phrases were conducted to investigate whether the preference was due to a syntactic impairment for mass nouns or a specific deficit of the determiner “some.” The noun phrases were composed of either the determiner “a” and a singular count noun or the determiner “some” and a plural count or a mass noun. Herbert and Best predicted that a deficit restricted to the lexical item “some,” should cause problems in the production of phrases with both mass nouns and plural count nouns. A syntactic deficit for mass nouns however, should lead exclusively to problems in the production of mass noun phrases. The results revealed, once again, significantly better performance for singular count nouns than for mass nouns in reading aloud and repetition. The errors for singular count nouns consisted mainly of omissions and substitutions of the determiner “a” by “the.” However, MH tended to omit the determiner “some” for all mass and most of the plural count nouns. Thus, MH's performance supported a deficit of the determiner “some,” rather than a mass noun impairment. MH's determiner deficit can be accounted for by an impairment of specific determiner lemma nodes (e.g., some, much) and/or the links from these specific determiner lemma nodes to their lexical-syntactic attributes (e.g., [mass], [plural]). As a result of such an impairment, activation which is sent from noun lemma nodes to lexical-syntactic attributes and forwarded to the affected determiner lemma nodes would not be sufficient for the determiner's selection. The retrieval of the determiners “a” and “the” could have been unimpaired, due to their higher frequency. Overall, MH's determiner specific deficit can be explained in two of the possible extension of Levelt et al.'s theory described above (Count And Mass Marked, the Count Unspecified Mass Marked hypotheses). However, in addition to her determiner problems in noun phrase and sentence production, MH had a deficit in naming bare mass nouns. Similar to CN, MH's countability specific bare noun deficit could be explained through an impairment of semantic features that are critical for the activation of mass nouns (e.g., UNINDIVIDUATED, NON-ATOMIC). Herbert and Best showed that MH's performance on mass nouns improved when the syntactic cue “some” was provided. The auditory presentation of “some” would activate its determiner lemma node which in turn, via its conceptual-semantic representation(s) (e.g., UNINDIVIDUATED) would activate noun lemma nodes which comprise this feature (i.e., many mass nouns). Hence, for MH, the determiner “some” facilitated the selection of mass noun lemma nodes by virtue of shared semantic representation(s).

## Discussion

In this review, we first specified the characteristics of mass and count nouns and discussed ideas regarding the basis of their differences in semantics and syntax. Theoretical accounts of mass and count noun processing were introduced (Levelt, 1989; Levelt et al., 1999; Barner and Snedeker, 2005, 2006; Taler and Jarema, 2006). These accounts were extended to provide potential mechanisms for processing of mass and count nouns in language production and comprehension using the theory of Levelt et al. (1999) as a basis. In the *Count and Mass Marked hypothesis*, countability information is hypothesized to be represented in form of two separate nodes, a [mass] node for mass nouns and a [count] node for count nouns, by analogy to Levelt et al.'s handling of grammatical gender. In the *Count Unspecified Mass Marked hypothesis*, derived from Taler and Jarema (2006), both count and mass nouns are represented by a countability node, mass nouns however are marked and possess an additional lexical-syntactic attribute [mass]. In the *Mass Unspecified Count Marked hypothesis* based on Barner and Snedeker (2005, 2006), count nouns are specified by a lexical-syntactic attribute [count] and a conceptual-semantic feature INDIVIDUATED and mass nouns remain syntactically and semantically unspecified.

We then presented research with normal speakers and language impaired speakers which delivered insights into the representation and processing of mass and count nouns. In most of these studies specific impairments and/or differential effects were found related to the manipulation of the categories of mass and/or count. Each of these experimental investigations also allowed us to evaluate the theoretical accounts.

Vigliocco et al. (1999) and Biedermann et al. (2008) showed that participants, given a definition, were able to retrieve lexical-syntactic information regarding the mass and count status of nouns in TOT states at rates greater than when not in TOT states. This supported the proposal that countability is represented at a lexical-syntactic (lemma) level. In addition, the fact that access of phonological information was found to be independent from lexical-syntactic information suggests that (at least partial) word form access can be achieved without selection of lexical-syntactic mass/count attributes. The fact that bare noun processing may proceed without selection of lexical-syntactic attributes of countability is also supported by Semenza et al.'s case FA, who showed an isolated impairment of mass nouns, but only when lexical-syntactic processing was required. Further support for a lexical-syntactic representation of countability information came from Steinhauer et al's (2001) ERP study which found an independent grammatically related frontal negativity effect during processing of mass and count nouns in sentences.

The countability effects which were found in the semantic categorization tasks (Bisiacchi et al., 2005) can be argued to be based on semantic differences between mass and count noun referents. Mondini et al. (2008) suggested that count noun referents are semantically more concrete possibly by representing individuated objects with clear boundaries. While meanings of mass nouns are semantically more abstract (or less concrete) possibly by representing unindividuated substances/aggregates without clear boundaries. The distinction between semantically more concrete vs. abstract representations has been often explained by a difference in their semantic richness (Allport, 1985; Breedin et al., 1994; Strain et al., 1995). Semantic richness can be defined by the number of semantic features with concrete words having more semantic features than abstract words (Plaut and Shallice, 1993; Strain et al., 1995). Hence according to the “number of features” account, at least some meanings of mass nouns could be less concrete due to their relatively lower number of semantic features (e.g., milk: white, liquid, creamy, comes from cows; rice: white/brown/black, small grains, grows in Asia) compared to the meanings of count nouns (e.g., cat: animal, pet, purrs, has whiskers, has a long tail, has four legs, has fur, catches mice, dislikes dogs etc.). Consequently, count nouns could be easier to categories as their semantic representation is richer and therefore more explicit than the semantic representation of mass nouns. Further evidence for the existence and influence of differences in semantic representations on mass and count noun processing was found in tasks like grammatical judgments and lexical decision with morphological priming (Gillon et al., 1999; El Yagoubi et al., 2006): Processing was slowed down in the presence of semantic features which were atypical for mass or count noun referents (e.g., atomic mass noun referents). The number of features account and the assumption of differences in features for mass and count noun referents require decomposed representations in form of semantic features at the conceptual-semantic level. Even though, Levelt et al. (1999) propose that word meanings are represented non-decompositionally, they do assume the existence of some semantic features such as MULTIPLE for plural nouns. Hence, it is not entirely implausible to propose semantic features such as INDIVIDUATED and UNINDIVIDUATED at the conceptual-semantic level which are activated and selected for mass and/or count noun referents.

Our extended version of Levelt et al.'s theory, is also able to explain some of the countability specific impairments in aphasia in either of the theories where mass nouns are marked by a lexical-syntactic attribute (*Count And Mass Marked hypothesis, Count Unspecified Mass Marked hypothesis*) but not when mass nouns are unmarked (as in the *Mass Unspecified Count Marked hypothesis*). FA's (Semenza et al., 1997) mass noun deficits in lexical-syntactic tasks can be accounted for by an impairment of the [mass] node at the lexical-syntactic level. MH's (Herbert and Best, 2010) determiner specific deficit can be explained by an impairment of specific determiner lemma nodes and/or the links from determiner lemma nodes to the lexical-syntactic attributes [mass] and [plural]. MH's difficulties in naming bare mass nouns can be explained with the conceptual-semantic account in which mass and count nouns differ in terms of semantic features at the conceptual-semantic level (e.g., mass nouns: UNINDIVIDUATED, NON-ATOMIC). Hence, an impairment of these features could either result in difficulties in naming mass nouns like for MH, or in naming count nouns as in the case of CN (Semenza et al., 2000). Considering the number of features account, MH's bare noun difficulties could also be accounted for by a general semantic impairment which would affect mass nouns more than count nouns as their semantic representations tend to be underspecified, or less rich compared to count nouns.

The different patterns across people with aphasia may also be due to a number of additional factors. These include the relative proportions of mass and count nouns in the lexicon and hence the frequency with which semantic mass and count noun related features are activated. For example, English has many more count than mass nouns (Brown and Berko, 1960; Baayen et al., 1995; Iwasaki et al., 2010), which could lead to more frequent activation of count specific features (e.g., INDIVIDUATED, COUNTABLE) compared to mass specific features. Another factor which could affect lexical-syntactic processing of mass and count noun phrases is the frequency of co-occurrence between mass/count nouns and specific determiners/quantifiers in a mass and count marked context. Further research is required to investigate in how far these factors influence mass/count processing.

**Figure 11:**
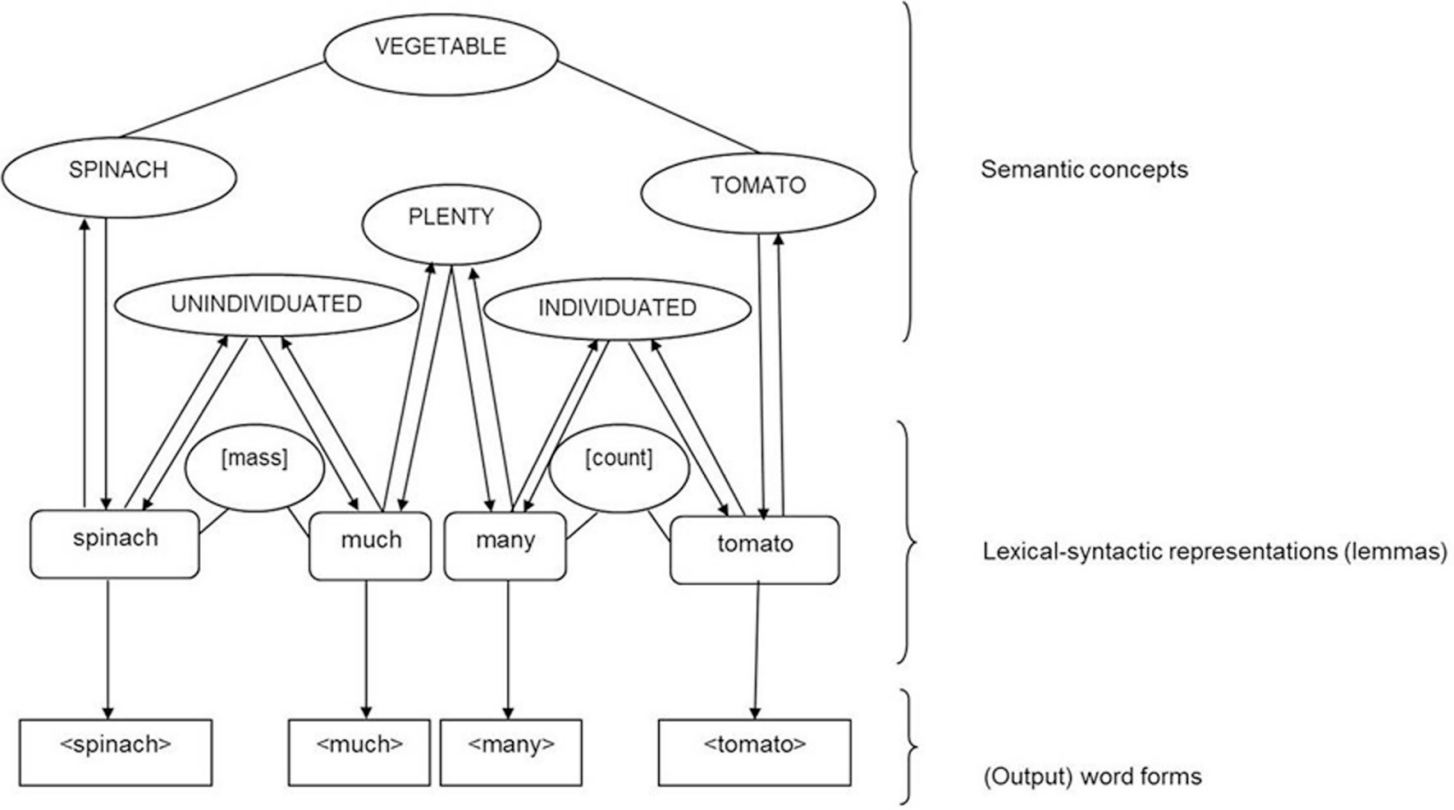
**Illustration of the processing and representation of mass nouns (e.g., spinach) and count nouns (e.g., tomato) in a theory of language production derived from Levelt et al.'s (1999) theory, with separate count and mass properties at the lexical-syntactic (lemma) level, and semantic concepts/features INDIVIDUATED for count nouns and count noun determiners and UNINDIVIDUATED for mass nouns and mass noun determiner at the conceptual-semantic level**.

In summary, the experimental evidence suggests that mass and count nouns are both specified at the lexical-syntactic level for countability under an account we have labeled the Count and Mass Marked account. However, it also appears that conceptual-semantic differences between mass and count nouns can influence processing. We therefore incorporate conceptual-semantic differences within the Count and Mass Marked account (see Figure 11). While our extended version of Levelt et al.'s (1999) theory of language production can explain the empirical findings of mass and count noun processing presented in this review, we do not claim that it is the only possible theoretical account. The patterns of semantic and lexical-syntactic processing of mass and count nouns could possibly also be accounted for in a more interactive theory of language processing, for example the interactive activation model by Dell (1986, 1990) and Dell et al. (1997). However, to derive and test predictions from such a theory, computational implementation would be needed, which is beyond the scope of this review.

Finally, we will briefly address how nouns which are frequently used as both mass and count nouns, so called dual nouns, are represented within our extended version of Levelt et al.'s (1999) theory. As we mentioned in the introduction, some dual nouns, such as “chicken” and “lamb,” not only behave syntactically differently but have also a different meaning depending on whether they are mass or count nouns (mass meaning: meat; count meaning: the animal). These kind of dual nouns are likely to be represented in a similar way to homophones (or polysemes) with one word form but two lemma nodes at the lexical-syntactic (lemma) level (and two concepts). Each of the two lemma nodes is connected to different lexical-syntactic attributes (e.g., mass or count). Depending on the speaker's intention, one of the two lexical-concepts of a dual noun would be selected and send activation to either the mass or count specific lemma node (see for homophone representation: Jescheniak and Levelt, 1994; Caramazza et al., 2001; Miozzo and Caramazza, 2005; Biedermann and Nickels, 2008a,b).

However, it is also the case that some nouns which are almost exclusively used as either mass or count (e.g., honey, dog), can nevertheless be flexibly used as mass or count nouns (e.g., the shop stocks many different honeys; there was dog all over the road) depending on the conceptual/perceptual characteristics to which a speaker intends to refer (Allan, 1980; Wisniewski et al., 2003). This supports a conceptual-semantic difference in the speakers' understanding of the characteristics a prototype mass or count noun should possess. It is also in line with the experimental evidence which shows that mass and count nouns are not only syntactically but also semantically specified for countability. The semantic specification can be assumed to be more flexibly used by speakers, whereas the lexical-syntactic specification of these nouns remains categorical. Hence, when a speaker wants to emphasize certain mass like characteristics in a count noun, relevant features (e.g., INDIVIDUATED, COUNTABLE) could become activated at the conceptual-semantic level, resulting in an overriding of the standard syntactic specification and the use of a count noun in a mass noun context with mass noun determiners.

Frisson and Frazier (2005) propose an alternative theory where, by default, nouns are lexical-syntactically specified for being either mass or count. These default or underived forms are also conceptual-semantically specified with mass nouns denoting a substance and count nouns representing an individuated entity. However, the mass/count status of a word can be changed through the application of lexical rules which results in derived forms. Mass nouns can be turned into count nouns through a proportioning rule (e.g., some beer vs. three beers), and count nouns into mass nouns through a grinding rule (e.g., three pears vs. a small amount of pear). These accounts provide potential explanations for the flexible use of mass and count nouns.

## Conclusion

Countability is one of the fundamental distinctions in the grammatical categorization of nouns in many languages and relates to the perception and semantic representation of objects. This review has discussed how mass/count information might be represented considering empirical findings in the literature, including studies of countability with language unimpaired and impaired speakers in both language comprehension and production. As a result, we proposed an explicit architecture for mass/count representation at the lexical-syntactic and conceptual-semantic levels which can account for current empirical data. We hope that the proposed model can further assist future experimental and computational studies with the process of formulating and testing predictions, and facilitate understanding of patterns of language behavior and breakdown related to both countability and to other lexical-syntactic properties/features.

## Author note

During the preparation of this paper, Nora Fieder was funded by a Macquarie University Research Excellence (MQRES) scholarship, Lyndsey Nickels was funded by an Australian National Health and Medical Research Council Senior Research Fellowship and an Australian Research Council Future Fellowship and Britta Biedermann by an ARC Australian Post-Doctoral Fellowship.

### Conflict of interest statement

The authors declare that the research was conducted in the absence of any commercial or financial relationships that could be construed as a potential conflict of interest.

## Footnotes

^1^Salsify is a plant in the genus Tragopogon. Salsify can grow to 60 cm height. Its stem is unbranched and the leaves are grasslike. The color of the flower can be purple, yellow or bronze. The roots and shoots of salsify can be eaten raw or cooked. The taste is described as being sweet and similar to oysters. Very popular in France and Italy it is used as an accompaniment to meats and in soufflés and is a very popular snack in Belgium served as a fritter with beer.

^2^Middleton et al. (2004, p. 372) defined aggregates as “collections of relatively small, homogenous entities” (e.g., rice, gravel, confetti, sand).

^3^Middleton et al. (2004) describe “non-individuated entity” as a term which is more abstract than the term substance and comprises more kinds of mass entities. Hence, in addition to substances it refers also to cognitive events (e.g., anger), physical events (e.g., sleep) and sounds (e.g., thunder).

^4^The term “bare nouns” is used in this paper to describe nouns which appear in isolation (e.g., water) instead of as part of a noun phrase with a determiner and/or adjective (e.g., the water, cold water).

^5^Semantic countability information of nouns/noun phrases can also influence linguistic properties of the verbs or verb phrases, such as telicity (Verkuyl, 1972). Verbs/verb phrases representing an event or action which can be completed in the sense that they have linguistically seen an endpoint are referred to as telic. While verbs/verb phrases which represent an event/action which cannot be completed as they have linguistically seen no limit or endpoint are referred to as atelic. The telicity of verbs has been suggested to relate to the countability (or ability to quantify) nouns which serve as direct objects in the sentence. For example, the sentence “Marie ate rice.” has no endpoint as the amount of rice is not limited hence the object's mass noun characteristic of being uncountable leads to an atelic reading of the verb. In comparison a sentence including the same verb but a singular count noun “Marie ate an apple.” or the same noun but combined with a unit of measurement ‘Marie ate a spoon of rice.’ leads to a telic verb reading in which the event has an endpoint. This endpoint is determined by the verb type (e.g., “eat” and “build” are dynamic verbs which change the state of the object noun, for example “eating” reduces the quantity of food) and the countability of the noun/noun phrase in object position (e.g., the ability to quantify the object represented by the noun/noun phrase (Krifka, 1989), for example “a spoon of rice” is a limited amount of food which can be reduced to zero by eating it). This is one of several linguistically based semantic accounts (e.g., Verkuyl, 1972; Krifka, 1989). There are also a number of linguistically based syntactic accounts (e.g., Borer, 1994) (see also for a review of the different semantic and syntactic accounts MacDonald, 2010) which would however exceed this review.

^6^There are no reliable cues to countability from either phonology or morphology (while bare plural count nouns are marked, singular count and mass nouns cannot be distinguished morphologically).

^7^Even though grammatical gender is a grammatically derived and hence a fixed lexical-syntactic property, in some cases its selection can be influenced by conceptual-semantic information. For example, Schiller et al. (2003) found that participants made faster gender decisions for words which have biological sex (e.g., die_fem_ Frau_fem_ − the woman) and are congruent regarding their grammatical and biological gender compared to words with no biological sex (e.g., der_masc_ Tisch_masc_ – the table) (see also Nickels et al., 2014). Further evidence for an influence of biological gender on lexical-syntactic processing was found by Vigliocco and Franck (1999), (2001). In a sentence completion tasks with gender marked adjective noun phrases, Italian and French speakers made fewer agreement errors between adjective and head noun for nouns which also had biological gender.

^8^Although it is possible that the singular nodes for mass and count nouns are different nodes, this seems unlikely given the general assumption of Levelt et al.'s (1999) theory.

## References

[B1] Allan K. (1980). Nouns and Countability. Language 56, 541–547 10.2307/41444924801445

[B2] Allport D. A. (1985). “Distributed memory, modular systems and dysphasia,” in Current Perspectives in Dysphasia, eds Newman S. K. Epstein R. (Edinburgh: Churchill Livingstone), 30–60

[B3] Armon-Lotem S. Crain S. Varlokosta S. (2004). Interface conditions in child language: cross-linguistic studies on the nature of possession. Lang. Acquis. 12, 171–217 10.1080/10489223.1995.9671743

[B4] Baayen R. H. Burani C. Schreuder R. (1996). “Effects of semantic markedness in the processing of regular nominal singulars and plurals in Italian,” in Yearbook of Morphology, eds Booij G. E. Marle J. V. (Dordrecht: Kluwer Academic), 13–33

[B5] Baayen R. H. Dijkstra T. Schreuder R. (1997). Singulars and plurals in Dutch: evidence for a parallel dual-route model. J. Mem. Lang. 37, 94–117 10.1006/jmla.1997.2509

[B6] Baayen R. H. Piepenbrock R. Gulikers L. (1995). The CELEX Lexical Database (CD-ROM). Philadelphia, PA: Linguistic Data Consortium. University of Pensylvania

[B8] Badecker W. Miozzo M. Zanuttini R. (1995). The two-stage model of lexical retrieval: evidence from a case of anomia with selective preservation of grammatical gender. Cognition 57, 193–216 10.1016/0010-0277(95)00663-J 8556841

[B9] Barner D. Snedeker J. (2005). Quantity judgements and individuation: evidence that mass nouns count. Cognition 97, 41–66 10.1016/j.cognition.2004.06.009 16139586

[B10] Barner D. Snedeker J. (2006). Children's early understanding of mass-count syntax: individuation, lexical content, and the number asymmetry hypothesis. Lang. Learn. Dev. 2, 163–194 10.1207/s15473341lld0203_2

[B11] Bates E. MacWhinney B. (1982). “Functionalist approaches to grammar,” in Language Acquisition: The State of the Art, eds Wanner E. Gleitman L. R. (Cambridge: Cambridge University Press), 173–218

[B12] Berg T. (1992). Prelexical and postlexical features in language production. Appl. Psycholinguist. 13, 199–235 10.1017/S0142716400005567

[B13] Biedermann B. Nickels L. (2008a).The representation of homophones: more evidence from the remediation of anomia. Cortex 44, 276–293 10.1016/j.cortex.2006.07.004 18387557

[B14] Biedermann B. Nickels L. (2008b). Homographic and heterographic homophones in speech production: does orthography matter? Cortex 44, 683–697 10.1016/j.cortex.2006.12.001 18472038

[B15] Biedermann B. Ruh N. Nickels L. Coltheart M. (2008). Information retrieval in tip of the tongue states: new data and methodological advances. J. Psycholinguist. Res. 37, 171–198 10.1007/s10936-007-9065-8 18046649

[B16] Bisiacchi P. Mondini S. Angrilli A. Marinelli K. Semenza C. (2005). Mass and count nouns show distinct EEG cortical processes during an explicit semantic task. Brain Lang. 95, 98–99 10.1016/j.bandl.2005.07.054

[B17] Bloomfield L. (1933). Language. New York, NY: Holt & Co

[B18] Bock K. Carreiras M. Meseguer E. (2012). Number meaning and number grammar in English and Spanish. J. Mem. Lang. 66, 17–37 10.1016/j.jml.2011.07.008

[B19] Bock K. Eberhard K. M. Cutting C. (2004). Producing number agreement: how pronouns equal verbs. J. Mem. Lang. 51, 251–278 10.1016/j.jml.2004.04.005

[B20] Bock K. Eberhard K. M. Cutting J. C. Meyer A. S. Schriefers H. (2001). Some attractions of verb agreement. Cogn. Psychol. 43, 83–128 10.1006/cogp.2001.0753 11527432

[B21] Bock K. Middleton E. L. (2011). Reaching agreement. Nat. Lang. Linguist. Theory 29, 1033–1069 10.1007/s11049-011-9148-y9814324

[B22] Borer H. (1994). “The projections of arguments,” in Functional Projections, eds Benedicto E. Runner J. (Amherst: GLSA), 19–47

[B23] Breedin S. D. Saffran E. M. Coslett H. (1994). Reversal of the concreteness effect in a patient with semantic dementia. Cogn. Neuropsychol. 11, 617–660 10.1080/0264329940825198718973766

[B24] Brown R. Berko J. (1960). Word association and the acquisition of grammar. Child Dev. 31, 1–14 10.2307/1126377 13805002

[B25] Caramazza A. (1997). How many levels of processing are there in lexical access? Cogn. Neuropsychol. 14, 177–208 10.1080/026432997381664

[B26] Caramazza A. Costa A. Miozzo M. Bi Y. (2001). The specific-word frequency effect: implications for the representation of homophones in speech production. J. Exp. Psychol. Learn. Mem. Cogn. 27, 1430–1450 10.1037/0278-7393.27.6.1430 11713878

[B27] Caramazza A. Miozzo M. (1997). The relation between syntactic and phonological knowledge in lexical access: evidence from the “tip-of-the tongue” phenomenon. Cognition 64, 309–364 10.1016/S0010-0277(97)00031-0 9426505

[B28] Caramazza A. Miozzo M. (1998). More is not always better: a response to Roelofs, Meyer, and Levelt. Cognition 69, 231–241 9894406 10.1016/s0010-0277(98)00057-2

[B29] Cheng L. L.-S. Sybesma R. (1998). Yi-wan Tang, Yi-ge Tang: classifiers and massifiers. Tsing Hua J. Chin. Stud. 28, 385–412

[B30] Cheng L. L.-S. Sybesma R. (1999). Bare and not-so-bare nouns and the structure of NP. Linguist. Inq. 30, 509–542 10.1162/002438999554192

[B31] Chierchia G. (1998). Reference to kinds across languages. Nat. Lang. Semantics 6, 339–405 10.1023/A:1008324218506

[B32] Chierchia G. (2010). Mass nouns, vagueness and semantic variation. Synthese 174, 99–149 10.1007/s11229-009-9686-6

[B34] Dell G. S. (1986). A spreading activation theory of retrieval in sentence production. Psychol. Rev. 93, 283–321 10.1037/0033-295X.93.3.283 3749399

[B35] Dell G. S. (1990). Effects of frequency and vocabulary type on phonological speech errors. Lang. Cogn. Process. 5, 313–349 10.1080/0169096900840706618587703

[B36] Dell G. S. Schwartz M. F. Martin N. Saffran E. M. Gagnon D. A. (1997). Lexical access in aphasic and nonaphasic speakers. Psychol. Rev. 104, 801–838 10.1037/0033-295X.104.4.801 9337631

[B38] Frisson S. Frazier L. (2005). Carving up word meaning: portioning and grinding. J. Mem. Lang. 53, 277–291 10.1016/j.jml.2005.03.004

[B39] Gao M. Malt B. (2009). Mental representation and cognitive consequences of Chinese individual classifiers. Lang. Cogn. Process. 24, 1124–1179 10.1080/01690960802018323

[B40] Garrard P. Carroll E. Vinson D. Vigliocco G. (2004). Dissociation of lexical syntax and semantics: evidence from focal cortical degeneration. Neurocase 10, 353–362 10.1080/13554790490892248 15788273

[B41] Gillon B. S. Kehayia E. Taler V. (1999). The mass/count distinction: evidence from online psycholinguistic performance. Brain Lang. 68, 205–211 10.1006/brln.1999.2081 10433760

[B101] Goldrick M. Rapp B. (2002). A restricted interaction account (RIA) of spoken word production: the best of both worlds. Aphasiology 16, 20–55 10.1080/02687040143000203

[B42] Herbert R. Best W. (2010). The role of noun syntax in spoken word production: evidence from aphasia. Cortex 46, 329–342 10.1016/j.cortex.2009.03.016 19555932

[B43] Hillis A. E. Caramazza A. (1991). Category-specific naming and comprehension impairment: a double dissociation. Brain 114, 2081–2094 10.1093/brain/114.5.2081 1933235

[B44] Imai M. Gentner D. (1997). A cross-linguistic study on early word meaning. Universial ontology and linguistic influence. Cognition 62, 169–200 10.1016/S0010-0277(96)00784-6 9141906

[B45] Iwasaki N. Vinson D. P. Vigliocco G. (2010). Does the grammatical count/mass distinction affect semantic representations? Evidence from experiments in English and Japanese. Lang. Cogn. Process. 25, 189–223 10.1080/01690960902978517

[B46] Jackendoff R. (1991). Parts and boundaries. Cognition 41, 9–45 10.1016/0010-0277(91)90031-X 1790657

[B47] Jacobsen T. (1999). Effects of grammatical gender on picture and word naming: evidence from German. J. Psycholinguist. Res. 28, 499–514 10.1023/A:1023268310519 10453481

[B48] Jescheniak J. D. (1999). Gender priming in picture naming: modality and baseline effects. J. Psycholinguist. Res. 28, 729–737 10.1023/A:1023229329967

[B49] Jescheniak J. D. Levelt W. J. M. (1994). Word frequency effects in speech production: retrieval of syntactic information and of phonological form. J. Exp. Psych. Learn. Mem. Cogn. 20, 824–843 10.1037/0278-7393.20.4.8249894405

[B50] Krifka M. (1989). “Nominal reference, temporal constitution and quantification in event semantics,” in Semantics and Contextual Expressions, eds Bartsch R. van Benthem J. van Emde Boas P. (Dordrecht: Foris Publications), 75–115

[B51] Krifka M. (1995). “Common nouns: a contrastive analysis of Chinese and English,” in The Generic Book, eds Carlson G. Pelletier F. J. (Chicago: University of Chicago Press), 398–411

[B52] La Heij W. Mak P. Sander J. Willeboordse E. (1998). The gender-congruency effect in picture-word tasks. Psychol. Res. 61, 209–219 10.1007/s00426005002616569158

[B53] Langacker R. W. (1987). Nouns and verbs. Language 63, 53–94 10.2307/415384

[B54] Levelt W. J. M. (1989). Speaking: From Intention to Articulation. Cambridge, MA: MIT Press

[B56] Levelt W. J. M. Roelofs A. Meyer A. S. (1999). A theory of lexical access in speech production. Behav. Brain Sci. 22, 1–75 10.1017/S0140525X99001776 11301520

[B57] Liu F. (2012). The count-mass distinction of abstract nouns in Mandarin Chinese. UCLA Working Papers in Linguistics, Theories of Everything 17, 215–221

[B58] MacDonald J. E. (2010). The aspectual influence of the noun: (A)telicity, (A)symmetry, incrementality and universality. Lang. Linguist. Compass 4, 831–845 10.1111/j.1749-818X.2010.00227.x

[B59] MacNamara J. (1972). Cognitive basis of language learning in infants. Psychol. Rev. 79, 1–13 10.1037/h0031901 5008128

[B60] MacNamara J. (1982). Names for Things: A Study of Human Learning. Cambridge, MA: MIT Press

[B61] Meyer A. S. Bock J. K. (1999).Representation and processes in the production of pronouns: some perspectives from Dutch. J. Mem. Lang. 41, 281–301 10.1006/jmla.1999.2649

[B62] Middleton E. L. Wisniewski E. J. Trindel K. A. Imai M. (2004). Separating the chaff from the oats: evidence for a conceptual distinction between count noun and mass noun aggregates. J. Mem. Lang. 50, 371–394 10.1016/j.jml.2004.02.005

[B63] Miozzo M. Caramazza A. (2005). The representation of homophones: evidence from the distractor frequency effect. J. Exp. Psychol. Learn. Mem. Cogn. 31, 1360–1371 10.1037/0278-7393.31.6.1360 16393051

[B64] Mondini S. Angrilli A. Bisiacchi P. Spironelli C. Marinelli K. Semenza C. (2008). Mass and count nouns activate different brain regions: an ERP study on early components. Neurosci. Lett. 430, 48–53 10.1016/j.neulet.2007.10.020 18035489

[B65] Mondini S. Kehayia E. Gillon B. Arcara G. Jarema G. (2009). Lexical access of mass and count nouns. How word recognition reaction times correlate with lexical and morpho-syntactic processing. Ment. Lexicon 4, 354–379 10.1075/ml.4.3.03mon

[B66] Nickels L. Biedermann B. Fieder N. Schiller N. O. (2014). The lexical syntactic representation of number. Lang. Cogn. Neurosci. [Epub ahead of print]. 10.1080/23273798.2013.879191

[B68] Plaut D. C. Shallice T. (1993). Deep dyslexia: a case study of connectionist neuropsychology. Cogn. Neuropsychol. 10, 377–500 10.1080/0264329930825346911716831

[B69] Quine W. V. O. (1960). Word and Object. Cambridge, MA: MIT Press

[B70] Rapp B. Goldrick M. (2000). Discreteness and interactivity in spoken word production. Psychol. Rev. 107, 460–499 10.1037/0033-295X.107.3.460 10941277

[B71] Roelofs A. (1992). A spreading-activation theory of lemma retrieval in speaking. Cognition 42, 107–142 10.1016/0010-0277(92)90041-F 1582154

[B72] Roelofs A. (1993). Testing a non-decompositional theory of lemma retrieval in speaking: retrieval of verbs. Cognition 47, 59–87 10.1016/0010-0277(93)90062-Z 8482071

[B73] Roelofs A. (2003). “Modeling the relation between the production and recognition of spoken word forms,” in Phonetics and Phonology in Language Comprehension and Production: Differences and Similarities, eds Meyer A. S. Schiller N. O. (Berlin: Mouton), 115–158 10.1515/9783110895094.115

[B74] Schiller N. O. Caramazza A. (2002). The selection of grammatical features in word production: the case of plural nouns in German. Brain Lang. 81, 342–357 10.1006/brln.2001.2529 12081404

[B75] Schiller N. O. Münte T. F. Horemans I. Jansma B. M. (2003). The influence of semantic and phonological factors on syntactic decisions: an event-related brain potential study. Psychophysiology 40, 869–877 10.1111/1469-8986.00105 14986840

[B76] Schriefers H. (1993). Syntactic processes in the production of noun phrases. J. Exp. Psychol. Learn. Mem. Cogn. 19, 841–850 10.1037/0278-7393.19.4.841

[B77] Schriefers H. Jescheniak J. D. (1999). Representation and processing of grammatical gender in language production: a review. J. Psycholinguist. Res. 28, 575–600 10.1023/A:1023264810403

[B78] Schriefers H. Jescheniak J. D. Hantsch A. (2002). Determiner selection in noun phrase production. J. Exp. Psychol. Learn. Mem. Cogn. 28, 941–950 10.1037/0278-7393.28.5.941 12219800

[B79] Semenza C. Mondini S. Cappelletti M. (1997). The grammatical properties of mass nouns: an aphasia case study. Neuropsychologia 35, 669–675 10.1016/S0028-3932(96)00124-8 9153029

[B80] Semenza C. Mondini S. Marinelli K. (2000). Count and mass nouns: semantics and syntax in aphasia and Alzheimer's disease. Brain Lang. 74, 395–431

[B81] Shapiro L. P. Zurif E. Carey S. Grossman M. (1989). Comprehension of lexical subcategory distinctions by aphasic patients: proper/common and mass/count nouns. J. Speech Hear. Res. 32, 481–488 2779194 10.1044/jshr.3203.481

[B82] Soja N. N. Carey S. Spelke E. (1991). Ontological categories guide young children's inductions of word meaning: object terms and substance terms. Cognition 38, 179–211 10.1016/0010-0277(91)90051-5 2049905

[B83] Sonnenstuhl I. Huth A. (2002). Processing and representation of German –n plurals: a dual mechanism approach. Brain Lang. 81, 276–290 10.1006/brln.2001.2524 12081399

[B84] Steinhauer K. Pancheva R. Newman A. J. Gennari S. Ullman M. T. (2001). How the mass counts: an electrophysiological approach to the processing of lexical features. Cogn. Neurosci. Neuropsychol. 12, 999–1005 11303776 10.1097/00001756-200104170-00027

[B85] Strain E. Patterson K. Seidenberg M.S. (1995). Semantic effects in single-word naming. J. Exp. Psychol. Learn. Mem. Cogn. 21, 1140–1154 10.1037/0278-7393.21.5.1140 8744959

[B86] Taler V. Jarema G. (2006). On-Line lexical processing in AD and MCI: an early measure of cognitive impairment? J. Neurolinguist. 19, 38–55 10.1016/j.jneuroling.2005.07.002

[B87] Taler V. Jarema G. (2007). Lexical access in younger and older adults: the case of the mass/count distinction. Can. J. Exp. Psychol. 61, 21–34 10.1037/cjep2007003 17479739

[B88] Taler V. Jarema G. Saumier D. (2005). Semantic and syntactic aspects of the mass/count distinction: a case study of semantic dementia. Brain Cogn. 57, 222–225 10.1016/j.bandc.2004.08.050 15780454

[B89] Van Berkum J. J. A. (1997). Syntactic processes in speech production: the retrieval of grammatical gender. Cognition 64, 115–152 10.1016/S0010-0277(97)00026-7 9385868

[B90] Verkuyl H. (1972). On the Compositional Nature of the Aspects. Dordrecht: D. Reidel Publishing Company 10.1007/978-94-017-2478-4

[B91] Vigliocco G. Antonini T. Garrett M. F. (1997). Grammatical gender is on the tip of Italian tongues. Psychol. Sci. 8, 314–317 10.1111/j.1467-9280.1997.tb00444.x

[B92] Vigliocco G. Franck J. (1999). When sex and syntax go hand in hand: gender agreement in language production. J. Mem. Lang. 40, 455–478 10.1006/jmla.1998.2624

[B93] Vigliocco G. Franck J. (2001). When sex affects syntax: contextual influences in sentence production. J. Mem. Lang. 45, 368–390 10.1006/jmla.2000.2774

[B94] Vigliocco G. Hartsuiker R. J. (2002). The interplay of meaning, sound, and syntax in sentence production. Psychol. Bull. 128, 442–472 10.1037/0033-2909.128.3.442 12002697

[B95] Vigliocco G. Lauer M. Damian M. F. Levelt W. J. M. (2002). Semantic and syntactic forces in noun phrase production. J. Exp. Psychol. Learn. Mem. Cogn. 28, 46–58 10.1037/0278-7393.28.1.46 11831212

[B96] Vigliocco G. Vinson D. P. Martin R. C. Garrett M. F. (1999). Is “Count” and “Mass” information available when the noun is not? an investigation of tip of the tongue states and anomia. J. Mem. Lang. 40, 534–558 10.1006/jmla.1998.2626

[B97] Warrington E. K. Crutch S. J. (2005). The semantic organisation of mass/count nouns and the representational locus of the mass/count distinction. Brain Lang. 95, 90–91 10.1016/j.bandl.2005.07.050

[B98] Wierzbicka A. (1988). The Semantics of Grammar. Amsterdam: John Benjamins 10.1075/slcs.18

[B99] Wisniewski E. J. Lamb C. A. Middleton E. L. (2003). On the conceptual basis for the count and mass noun distinction. Lang. Cogn. Process. 18, 583–624 10.1080/0169096034400004418206904

[B100] Yagoubi R. E. Mondini S. Bisiacchi P. Chiarelli V. Angrilli A. Semenza C. (2006). The electrophysiological basis of mass and count nouns processing. Brain Lang. 99, 8–219 10.1016/j.bandl.2006.06.10811303776

